# Estimating the introduction time of highly pathogenic avian influenza into poultry flocks

**DOI:** 10.1038/s41598-020-68623-w

**Published:** 2020-07-24

**Authors:** Peter H. F. Hobbelen, Armin R. W. Elbers, Marleen Werkman, Guus Koch, Francisca C. Velkers, Arjan Stegeman, Thomas J. Hagenaars

**Affiliations:** 1 Wageningen Bioveterinary Research, Houtribweg 39, 8221 RA Lelystad, The Netherlands; 2 Department of Farm Animal Health, Faculty of Veterinary Medicine, Utrecht University, Utrecht, The Netherlands; 3 Julius Centre for Health Sciences and Primary Care, University Medical Centre Utrecht, Utrecht, The Netherlands

**Keywords:** Viral infection, Viral infection

## Abstract

The estimation of farm-specific time windows for the introduction of highly-pathogenic avian influenza (HPAI) virus can be used to increase the efficiency of disease control measures such as contact tracing and may help to identify risk factors for virus introduction. The aims of this research are to (1) develop and test an accurate approach for estimating farm-specific virus introduction windows and (2) evaluate this approach by applying it to 11 outbreaks of HPAI (H5N8) on Dutch commercial poultry farms during the years 2014 and 2016. We used a stochastic simulation model with susceptible, infectious and recovered/removed disease stages to generate distributions for the period from virus introduction to detection. The model was parameterized using data from the literature, except for the within-flock transmission rate, which was estimated from disease-induced mortality data using two newly developed methods that describe HPAI outbreaks using either a deterministic model (A) or a stochastic approach (B). Model testing using simulated outbreaks showed that both method A and B performed well. Application to field data showed that method A could be successfully applied to 8 out of 11 HPAI H5N8 outbreaks and is the most generally applicable one, when data on disease-induced mortality is scarce.

## Introduction

Highly pathogenic avian influenza (HPAI) virus may cause severe clinical signs in poultry species, resulting in very high mortality rates^1–3^. Wild waterfowl is the main reservoir for low pathogenic avian influenza (LPAI) viruses^4^, which may evolve into highly pathogenic strains after introduction of LPAI subtypes H5 and H7 into poultry flocks^5,6^. Since the beginning of the 21th century, evidence has emerged that an endemic situation of HPAI virus strains in poultry flocks in South East Asia has created the opportunity for spill over into wild bird populations and migratory waterfowl is thought to be responsible for the spread of different HPAI viruses of subtype H5Nx to parts of the world where there is no endemic HPAI situation in commercial poultry^4^. For example, in the Netherlands recent outbreaks of H5N8 (in 2014 and 2016) and H5N6 (2017) in commercial poultry are thought to have been caused by virus that is brought by migratory waterfowl infected with HPAI to areas in the neighbourhood of poultry farms^7–9^. It is therefore important to be prepared for new HPAI outbreaks and have plans in place to control the spread of HPAI.

Estimating a time window for the introduction of a virus on a farm is important for the prevention and control of disease outbreaks for several reasons. Firstly, contact-tracing may be an important control measure that aims to prevent or reduce the size of an epidemic^10^. For this purpose (amongst others), many countries have put systems in place to record the location of farms and movement of poultry^11^. Having accurate estimates of the time of virus introduction will improve the efficiency and efficacy of contact tracing by focusing efforts on the time window that an outbreak farm was infectious. Secondly, the time from the introduction of a virus on a farm until its detection is a sensitive parameter in simulation models that try to estimate the size and duration of epidemics given various control measures^12,13^. Thirdly, it may also help to identify and assess the importance of potential risk factors for the introduction of a disease on a farm. For example, movements of people, vehicles or materials or ecological/environmental changes in the direct surroundings of the poultry house are more likely to be associated with virus introduction onto a farm if they happen close to the estimated introduction time^14^. And having accurate estimates of the time of disease introduction makes it possible to account for the timing of introduction through the year (e.g. the month) in the analysis of risk-factors for disease introductions.

Surprisingly, literature on the estimation of the introduction time of avian influenza into farms from outbreak data is scarce. To our knowledge there are three studies estimating the introduction time of avian influenza virus, two for LPAI and one for HPAI. The studies on LPAI derive the virus introduction time by fitting a deterministic simulation model to data on either the prevalence of infected and seroconverted animals^15^ or from data on egg production^16^. The study on HPAI describes a method to estimate the virus introduction time from disease-induced mortality data^17^. In the latter study, a stochastic simulation model was derived to describe the spread of HPAI virus within flocks of chickens. Using this model, a window for the introduction of HPAI was derived by tracking the time that was needed for the predicted disease-induced mortality to match the observed mortality at the time of detection.

The estimated time from HPAI virus introduction until detection will depend on the assumed value for the transmission rate, which represents the number of susceptible birds that is infected by an infectious bird during a given time interval. The transmission rate of HPAI virus may depend on many factors such as the HPAI virus subtype and clade, the poultry species, the farm type and the design of farm-buildings^5,18,19^. To our knowledge, there is only one published method to estimate a farm-specific between-bird transmission rate of HPAI virus from daily disease-induced mortality data^20,21^ (see “Methods”) and this method has a number of important limitations. First of all, it may overestimate the transmission rate, because it does not account for the fast increase in the number of infected individuals during even small time steps in the exponential phase of an outbreak. Secondly, this method can often not be applied to outbreaks for which disease-induced mortality data is only available for a few days (see the “Results” section of this study). This is an important limitation, because HPAI outbreaks, especially after detection of the first outbreak in a country and resulting in a high level of alertness, are often detected shortly after the disease-induced mortality exceeds the background mortality.

The aim of this paper is therefore to develop a more generally applicable approach for estimating a time window for the introduction time of HPAI virus into commercial poultry farms that (1) uses more accurate estimates of the farm-specific transmission rate and (2) can be applied to cases for which daily mortality is scarce. To show how this approach can be used in practice, we will apply it to HPAI-outbreaks on commercial poultry farms in the Netherlands during the years 2014 and 2016.

## Methods

### Data

We analysed mortality data from poultry farms in the Netherlands with outbreaks of HPAI virus clade 2.3.4.4 of subtype H5N8 during the years 2014 and 2016. The mortality of poultry in each house on these farms was recorded daily by the farmer on a production chart until detection of the disease and subsequent culling. Production charts with daily mortality were gathered by the Netherlands Food and Consumer Product Safety Authority (NVWA) during a standardized-investigation interview of the farmer after an outbreak was confirmed by laboratory diagnostics. In 2014 there were outbreaks on four chicken farms and one meat duck farm. In 2016 there were outbreaks on four chicken farms, four meat duck farms and on the premises of one backyard bird wholesale company. Chicken farms were either layers or broiler breeders. The outbreaks on one chicken farm and one duck farm in 2016 were excluded from our analysis, because disease-induced mortality could not be estimated for a period of at least 2 days (see below). The outbreak on the premises of the backyard bird wholesale company was also excluded, since mortality data were not available by species. Therefore, we analysed data from 11 out of the 14 HPAI H5N8 outbreaks in 2014 and 2016: 7 outbreaks on chicken farms and 4 outbreaks on duck farms. The virus introduction windows for these farms were estimated using mortality data for one specific poultry house per farm, where clinical disease signs were observed. The virus infections on most farms were also limited to one poultry house on the farm premises. For one outbreak on a duck farm in 2014 (farm E), enhanced mortality was observed in both an old and a young flock (kept in separate houses). We only used the data on the young flock, since the old flock was already slaughtered at the time of detection. For another outbreak on a duck farm in 2016 (farm H), enhanced mortality was detected in two houses and both houses were culled on the same day. However, only one of these houses was included in our analysis (house 1), because disease-induced mortality in the other house could not be estimated for a period of at least two days. For the two breeder farms, we combined mortality data for cocks and hens. Table 1 gives an overview of the characteristics of the outbreak farms. Daily mortality data were available for a period of a few weeks to several months prior to detection. The last day for which mortality was observed was usually one or two days before the culling date, but was the same as the culling date for two outbreak farms.

**Table 1:** Farm characteristics, disease-induced mortality and the estimated date of virus introduction for outbreaks of highly pathogenic avian influenza of subtype H5N8 on poultry farms in the Netherlands in 2014 and 2016.

Farm identifier	Farm type^a^	# Birds in infected house	Estimated cumulative mortality due to disease	Date last recorded mortality	Estimated date of virus introduction^b^
**Chickens 2014**					
A	Layer	23,459	1519	16/11/2014	01/11/2014
B	Layer	27,840	997	20/11/2014	10/11/2014
C	Layer	28,417	106	29/11/2014	17/11/2014
D	Broiler breeder	6,141	210	20/11/2014	–^c^
**Ducks 2014**					
E	Meat duck	14,500	115	21/11/2014	–^c^
**Chickens 2016**					
F	Layer	23,699	656	12/12/2016	05/12/2016
C	Layer	27,369	127	24/12/2016	18/12/2016
G	Broiler breeder	12,296	224	20/12/2016	–^c^
**Ducks 2016**					
H	Meat duck	7,800	83	01/12/2016	16/11/2016
I	Meat duck	8,550	416	01/12/2016	21/11/2016
E	Meat duck	15,000	105	15/12/2016	26/11/2016

^a^All outbreaks were in flocks that were housed indoors.

^b^Based on the mean time of virus introduction with transmission parameter $\beta$ estimated using method A (see “Methods”).

^c^The value of the transmission parameter $\beta$ could not be estimated and therefore the time of virus introduction could not be back-calculated.

### Back-calculation approach

To back-calculate from mortality data the probable time window of virus introduction into an individual outbreak farm, we used a SEIR (susceptible-exposed-infectious-recovered) modelling approach consisting of the following steps:

1. Determine the time interval prior to detection during which the recorded mortality can be considered to reflect disease-induced mortality
2. Estimate the farm-specific transmission parameter $\beta$ from the pattern of daily disease-induced mortality in the interval above. This parameter represents the daily number of secondary infections by one infectious animal in a susceptible population. The transmission rate was determined for each farm separately, because it may depend on factors such as the farm type, design of the poultry house and farm management specifics. Other model parameters describing the distributions of the latent and infectious periods and the percentage of infected birds dying were assumed to be the same for farms housing the same species and taken from the literature.
3. Enter the above-mentioned parameter values into a stochastic model and repeatedly simulate the disease outbreak on a farm until the calculated cumulative disease-induced mortality exceeds the observed mortality throughout the time interval determined in step 1. Each simulation is started with one latent individual. The length of the simulated period is recorded for every simulation and used to construct a distribution for the virus introduction time window.
4. Perform a sensitivity analysis to uncertain parameters.

Below, these four steps are described in more detail. For step 2, we developed two new methods (A and B). Method A estimates $\beta$ by directly fitting the SEIR model to the deterministic growth in mortality. Method B estimates $\beta$ by fitting back-calculated data on the daily number of infectious and newly infected animals using a likelihood function which accounts for the stochastic nature of the early phase of disease outbreaks.

### The time interval of disease-induced mortality

To estimate for each outbreak farm from which day onwards the observed mortality reflected HPAI virus-induced mortality instead of background mortality due to other causes, we used a moving weekly average approach^22^. In short, this approach identifies the first instance when, for two consecutive days, the mortality is significantly higher than the daily mortality during the preceding week-long period. The number of consecutive time points ${n}_{d}$ during which the recorded daily mortality was estimated to reflect HPAI virus-induced mortality is given in Table 2 for the different outbreak farms.

**Table 2:** The number of data points that were available for the estimation of transmission parameter $\beta$ using methods A and B for outbreaks of highly pathogenic avian influenza of subtype H5N8 on Dutch poultry farms in 2014 and 2016.

Farm identifier	Number of days for which mortality was recorded	Number of days for which mortality was assumed to be disease-induced (${n}_{d}$)^a^	Length of the latent period (days)	Number of data points available for $\beta$ estimation using method B (${n}_{t}$)
**Chickens 2014**				
A	28	7	2	4
B	61	3	2	0
C	12	3	2	0
D	45	2	2	0
**Ducks 2014**				
E	19	4	1	2
**Chickens 2016**				
F	16	3	1	1
C	241	2	1	0
G	55	2	1	0
**Ducks 2016**				
H	42	5	1	3
I	25	3	1	1
E	22	2	1	0

^a^This is the same as the number of data points available for the estimation of the transmission parameter $\beta$ using method A.

### General modelling approach

In order to estimate the farm-specific transmission rate $\beta$ and back-calculate the point of virus introduction from disease-induced mortality data, we used a SEIR modelling approach. The poultry population was divided into susceptible ($S$), exposed ($E$), infectious (${I}_{\text{R}},{I}_{\text{D}}$) and recovered ($R$) compartments. To track the cumulative number of disease-induced deaths, we added an additional compartment $D$. The model distinguishes two alternative infectious stages ${I}_{R}$ and ${I}_{D}$ in order to accommodate for differences in the infectious period distribution between animals that die and animals that recover. A fraction ${f}_{D}$ of the individuals that become infectious enter the compartment of individuals that die from disease and the remainder (${1-f}_{D}$) enters the compartment of recovering individuals.

Model parameters describing the distributions of the latent and infectious periods and the percentage of infected birds dying were assumed to be the same for farms housing the same species and taken from the literature. Below we present two alternative methods to estimate a farm-specific transmission parameter $\beta$ (methods A and B) using the general SEIR modelling approach.

**Figure 1:**
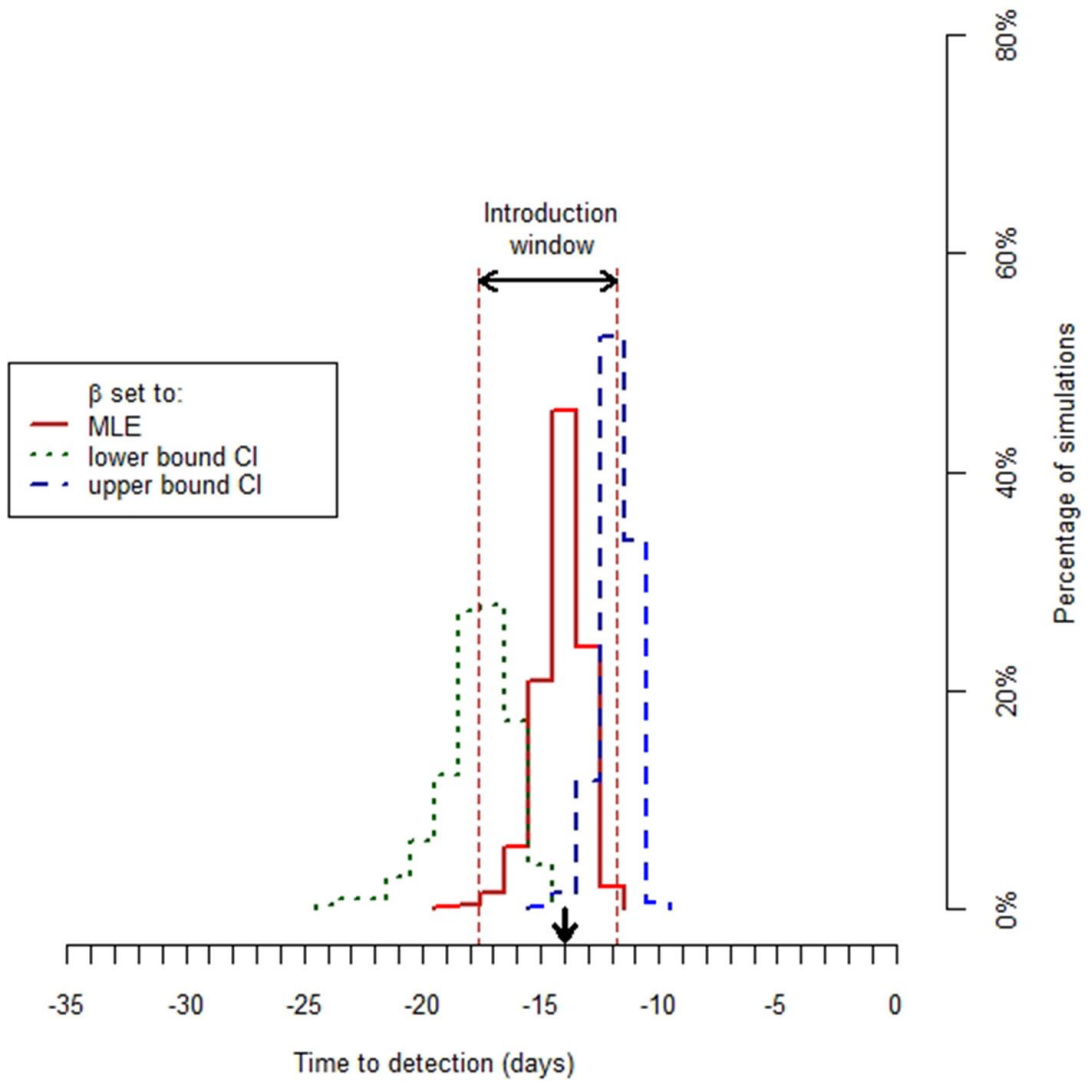
The distributions for the day of virus introduction on outbreak farm H for three different values of transmission parameter $\beta$ (maximum likelihood estimate (MLE), upper and lower 95% confidence bounds). Arrows indicate the introduction window and the mean time of virus introduction (see text).

Having obtained an estimate of transmission parameter $\beta$, we used a stochastic version of the general SEIR model (Supplementary Methods S1) to obtain a distribution for the time of disease introduction by repeatedly running this and recording the time interval from disease introduction until exceedance of the observed cumulative disease-induced mortality. The variation in outcome between the different model runs is due to the stochastic nature of the early phase of an outbreak, when the number of infectious individuals is still small. We define the ‘mean time of virus introduction’ as the mean of the distribution for the point of virus introduction with parameter $\beta$ set to its maximum likelihood estimate. The ‘introduction window’ is defined as the time interval between the means of the distributions for the point of virus introduction with parameter $\beta$ set to its lower and upper confidence bound, respectively (Fig. 1). The introduction window therefore provides a measure of the uncertainty in the time of virus introduction.

The distribution for the time of virus introduction for a given value of parameter $\beta$ was based on the output of 1,000 stochastic model runs. At the start of a simulation, one exposed animal was assumed to be present in an otherwise susceptible population. Simulations were discarded and did not count towards the 1,000 simulations, if the disease died out before the estimated cumulative disease-induced mortality at the time of detection was reached. The percentage of discarded simulations varied between outbreak farms from 0 to 28%, but was usually close to 0% (Supplementary Results S1). In the model simulations we used a fixed-time step of 0.01 day.

### Estimating model parameters that are not farm-specific

The length of the exposed stage, the infectious stages, the shape parameter of the gamma distribution for these stages and the fraction of animals dying from HPAI subtype H5N8 in 2014 were estimated from the literature for both chicken and duck farms. For the HPAI H5N8 outbreaks in 2016, these parameter values were estimated from a transmission experiment^23^. Further details about the methodology used for the literature review and the estimation of these parameters are described in Supplementary Methods S2. The default settings of epidemiological parameters other than $\beta$ are given in Table 3.

**Table 3:** The estimated values of epidemiological parameters for highly pathogenic avian influenza of subtype H5N8 that infected poultry farms in the Netherlands in 2014 and 2016.

Parameter	Estimates for the 2014 strain^a^	Estimates for the 2016 strain^a^	Sources for 2014 strain	Sources for 2016 strain
**Chickens**				
Length of latent period (days)	2	1	^41^	^23^
Length of infectious period (days)	2.5	1.1	^2,19,41–49^	^23^
% of infected animals dying from disease	100	100	^2,19,41–49^	^23^
Shape parameter of the gamma distribution for the latent and infectious periods	20	20	^2,41,45,46,48^	–^b^
**Ducks**				
Length of latent period (days)	1	1	^18^	^23^
Length of infectious period—survivors (days)	8.5	6	^18^	^23^
Length of infectious period—non-survivors (days)	8.5	3.5	^18^	^23^
% of infected animals dying from disease	20	20	^18^	^23^
Shape parameter of the gamma distribution for the latent and infectious periods	20	20	–^b^	–^b^

^a^See Supplementary Methods S2 for a description of the search terms and selection criteria that were used for the literature review and for a description of the estimation of parameter values from the literature data.

^b^In the absence of sufficient data, we used the value estimated for the distributions of the latent and infectious period of chickens in 2014.

### Estimating the farm-specific transmission rate

We used and compared two methods for the estimation of the transmission parameter *β* using a maximum likelihood approach. The likelihood-ratio test was used to obtain lower and upper 95% confidence bounds.

#### Method A

This method estimates the value of parameter $\beta$ by using a deterministic version of the general SEIR model and by fitting the model-predicted disease-induced mortality ${d}_{\text{pred}}$ to the observed mortality ${d}_{\text{obs}}$. We constructed the following expression for the likelihood $L$ assuming Poisson variation at each observation time point:

1 $$L={\prod }_{i=1}^{i={n}_{d}}\frac{{{(d}_{pred,i}}^{{d}_{obs,i}})({e}^{{-d}_{pred,i}})}{{d}_{obs,i}!}$$

Although this method requires the estimation of the time of virus introduction ($${t}_{intro})$$ in addition to transmission parameter $$\beta$$, the deterministic version of the SEIR model was only used to estimate the transmission parameter. As mentioned above, the virus introduction time was estimated in a subsequent step using the stochastic version of the SEIR to account for the stochastic nature of the initial phase of an outbreak. We used the deterministic version of the SEIR model to estimate parameter $$\beta$$ from disease-induced mortality data, because an outbreak will be in the exponential growth phase when mortality significantly increases.

A detailed description of the deterministic model is given in Supplementary Methods S3. Similar models consisting of ordinary differential equations (ODEs) with gamma distributed disease stages have previously been applied to describe the dynamics of LPAI^15^ and HPAI^24^ in poultry flocks. We numerically integrated the ODEs using a method which applies a variable step size in order to balance computational efficiency with a sufficient level of accuracy (function lsoda in R package deSolve).

#### Method B

Method B is building on the work by Tiensin et al.^21^ and Bos et al.^20^. Below, we first describe the method developed in these two studies and then explain why and how we adjusted it to obtain Method B. The original method uses a product-binomial likelihood function to account for the stochastic variation in the early phase of an outbreak when there are relatively low numbers of infected birds and the deterministic approximation of method A may be unsatisfactory. Analogous to the estimation of *β* from small-scale transmission experiments^3^, the method calculates the probability that susceptible individuals become infected or escape infection during one-day time steps. Denoting the number of time steps for which the probability on a certain number of new cases can be calculated as ${n}_{t}$, the likelihood function is given by:

2 $$L={\prod }_{i=1}^{i={n}_{t}}\left(\frac{S\left({t}_{i}\right)!}{C\left({t}_{i}+1\right)!\left(S\left({t}_{i}\right)-C({t}_{i}+1)\right)!}\right){({p}_{inf}({t}_{i}))}^{C({t}_{i}+1)}{(1-{p}_{inf}({t}_{i}))}^{S({t}_{i})-C({t}_{i}+1)}$$

Here ${p}_{inf}\left({t}_{i}\right)$ is the infection probability across the ith day. The number of new cases ($C$) and susceptible animals ($S$) need to be back-calculated from the observed disease-induced mortality as described elsewhere ^20,21^ and as summarized in Supplementary Methods S4. To be able to perform this back-calculation, the lengths of the latent and infectious periods must be rounded to a fixed integer numbers of days. The net number of data points (${n}_{t}$) that is available for estimating parameter $\beta$ equals to ${n}_{t}={n}_{d}-{l}_{E}-1$ with ${n}_{d}$ denoting the number of days for which disease-induced mortality was available (Supplementary Methods S4). A longer latent period decreases the number of data points (${n}_{t}$), because it reduces the overlap of the period for which the number of infectious individuals can be back-calculated with the period for which the number of new cases can be back-calculated.

According to a standard approximation^20,21^, the infection probability is approximated as ${p}_{inf}\left({t}_{i}\right)=1-{e}^{-\frac{\beta {I}_{\text{tot}}\left({t}_{i}\right)}{N\left({t}_{i}\right)}}$ with ${I}_{\text{tot}}\left({t}_{i}\right)$ and $N\left({t}_{i}\right)$ denoting the number of infected animals and the number of birds alive at time ${t}_{i}$. The infection hazard $\frac{\beta {I}_{\text{tot}}}{N}$ is assumed to remain constant during a one-day time step. However, the typically sharp rise in mortality in HPAI affected flocks suggests that the number of infectious individuals and therefore the infection hazard may significantly increase during a one-day time interval. To address this, we developed a new approximation to the likelihood by assuming exponential growth of the number of infectious individuals during the one-day time steps. This leads to the following equation for the probability of infection (see Supplementary Methods S5):

3 $${p}_{inf}(t)=1-{e}^{-\frac{\beta {I}_{tot}(t)({e}^{r}-1)}{{N}_{0}r}}$$

The value of growth rate $r$ can be calculated from the value of parameter $\beta$, the lengths of the exposed and infectious stages and the fraction of individuals dying from disease (if applicable) using the following equation (see Supplementary Methods S5):

4 $$r=\beta \left({f}_{D}{l}_{ID}+(1-{f}_{D}){l}_{IR}\right){e}^{-r{l}_{E}}\left(\frac{{f}_{D}}{{l}_{ID}}\left(1-{e}^{-r{l}_{ID}}\right)+\frac{{(1-f}_{D})}{{l}_{IR}}\left(1-{e}^{-r{l}_{IR}}\right)\right)$$

Here we use the total population size at the start of an outbreak ($${N}_{0})$$ as a close approximation of the actual number of birds alive, since usually less than 10% of a flock has been infected at the time of detection in the Netherlands.

### Goodness of fit

We used Lin's concordance correlation coefficient^26^ as a measure for the goodness of the model fit to the data. It  was calculated using the model-predicted vs. observed mortality counts for method A and using the model-predicted vs. back-calculated newly infected animals per day for method B.

### Model testing

We tested the accuracy of methods A and B as well as the method described in literature^20,21^ by applying them to simulated HPAI-outbreaks on chicken farms and by subsequently comparing the estimated values of the transmission rate $\beta$ with the value that was used to generate the outbreaks. For this purpose, we created outbreaks with transmission rates varying from 2.5 to 10 in steps of 2.5. In step 1 of our back-calculation approach, we assume all mortality to be disease-induced when the daily mortality exceeds a farm-specific threshold (see above). To explore the effect of this assumption, we created outbreaks with and without background mortality for each value of the transmission rate. If accounted for, the daily mortality rate was set to 0.02% and equals the average daily mortality on the 7 chicken farms in this study prior to the estimated time of disease introduction. For each combination of transmission and background mortality rate, we simulated one outbreak using the deterministic version of the general SEIR model (Supplementary Methods S3) and 200 outbreaks with the stochastic version of the general SEIR model (Supplementary Methods S1). The other parameter settings were the same for both models with a 1-day latent period, a 2-day infectious period, 100% mortality and the shape parameter of the gamma-distribution for the latent and infectious periods was set to 20. All simulations started with 1 latent individual in a poultry house with in total 25,000 birds. Parameter $\beta$ was fitted to the predicted daily disease-induced mortality. The average 97.5% percentile of the daily mortality on the 7 chicken farms in this study amounted to 0.06%. We therefore assumed that the daily disease-induced mortality must exceed 15 (0.06% of 25,000) to be flagged as disease-induced. Disease-induced mortality was assumed to be available for 4 days after disease-induced mortality exceeded the daily mortality threshold (Supplementary Methods & Results S1).

### Time of virus introduction and the introduction window

Using the default parameter settings (Table 3), we tried to estimate the parameter $\beta$ for each outbreak farm using both methods A and B. We subsequently determined the mean time of virus introduction and introduction window, as described above.

### Sensitivity and elasticity analysis

We determined the sensitivity of the transmission parameter $\beta$ and the mean time of virus introduction to changes in (1) the number of days with disease-induced mortality and (2) in the default settings of epidemiological parameters. The number of days with disease-induced mortality was decreased and increased by one day compared to the default scenario, except when there were only two days with disease-induced mortality in the default scenario (Table 2). For these outbreak farms, we only increased the number of days with disease-induced mortality by one day.

The ranges over which epidemiological parameters were varied are given in Supplementary Methods & Results S2. To compare the effect of uncertainty in the different epidemiological parameters on transmission rate $\beta$ and the mean time of virus introduction, we calculated the elasticity ($e$)^25^. Only method A was used in our sensitivity analysis as it is the most generally applicable one of the two methods.

### Decision rules

In order to obtain a minimal level of accuracy, we only accepted estimates of transmission rate $\beta$ when the following requirements were met:

1. the likelihood was based on ≥ 2 data points (${n}_{d}\ge 2$ for method A and ${n}_{t}\ge 2$ for method B)
2. the shape of the likelihood profile allowed estimation of the maximum likelihood estimate as well as upper and lower confidence bounds for the transmission parameter $\beta$
3. the agreement between the observed and predicted disease-induced mortality as measured by Lin’s concordance correlation coefficient^26^ (${r}_{l}$) was ≥ 0.75.

We set the minimum number of data points to 2, because the estimated transmission rate may change considerably when increasing the number of data points from 1 to 2, while further increasing the number of data points to 3 or more often had less effect on the estimated value of the transmission rate (results not shown). In addition, from a practical point of view, often there are only 2 or 3 data points available for the estimation of the transmission rate. The second requirement avoids estimates of the transmission rate without an indication of the uncertainty. The third requirement ensures a minimum quality of the fit and is a strict criterion, since we are dealing with biological data and therefore complex and stochastic processes. The value of coefficient ${r}_{l}$ is highest (equal to 1) when all points on a scatter plot of the observed versus predicted disease-induced mortality lie on the 45-degree line through the origin (perfect model fit). Deviations from the slope or the intercept (origin) of the 45-degree line will decrease the value of ${r}_{l}$.

### Software

All analyses were coded and performed using R version 3.4.0 (The R Foundation for Statistical Computing, 2017).

## Results

### Descriptive statistics of the mortality data

In Table 1 we list the outbreak farms, their type, and the duration and size of virus-induced mortality, estimated using the moving average approach^22^. Notably, for 8 of the 11 farms, the estimated period with virus-induced mortality was two or three days long. For the other three outbreak farms this period was between 4 to 7 days long.

### Estimates of the latent and infectious period length

The values for these parameters and their sources as derived from literature are summarized in Table 3. These values, determined by experimental infection trials, reflect the difference in time scales between the strains with the 2016 strain having a shorter infectious period than the 2014 strain in both chickens and ducks and also a shorter latent period in chickens.

### Model testing

We tested the methods A and B as well as the literature method by applying them to simulated HPAI outbreaks on a chicken farm for values of transmission parameter $\beta$ varying from 2.5 to 10 and in absence and presence of background mortality (Supplementary Methods & Results S1). Method A as well as B performed well when applied to the deterministic outbreaks for all transmission and background mortality rate scenarios and deviations of the estimated transmission rate from the true value were less than 8%. The confidence intervals for parameter $\beta$ were wider for method A than for method B, since method A also requires the estimation of a second parameter (see above). Contrary to methods A and B, the literature method overestimated the value of transmission parameter $\beta$ by a factor 1.4 to 2.8. The same picture emerges when applying the different methods for estimating parameter $\beta$ to the simulated stochastic outbreaks. Again, the estimates for $\beta$ obtained by methods A and B were usually much closer to the actual value used for the model simulations (deviations 8 to 23.7%) than the estimates obtained by the literature method (deviations 13.6 to 104%). Interestingly, estimates of the transmission rate by methods A and B deviated further from the true value for stochastic than for deterministic outbreaks and were always less than the true value of parameter $\beta$. This is probably due to the importance of stochastic effects in the early phase of an outbreak which slow down the increase in the daily disease-induced mortality in comparison to an outbreak predicted by a deterministic simulation model (Supplementary Methods & Results S1).

### Transmission parameter ${\varvec{\beta}}$

**Figure 2:**
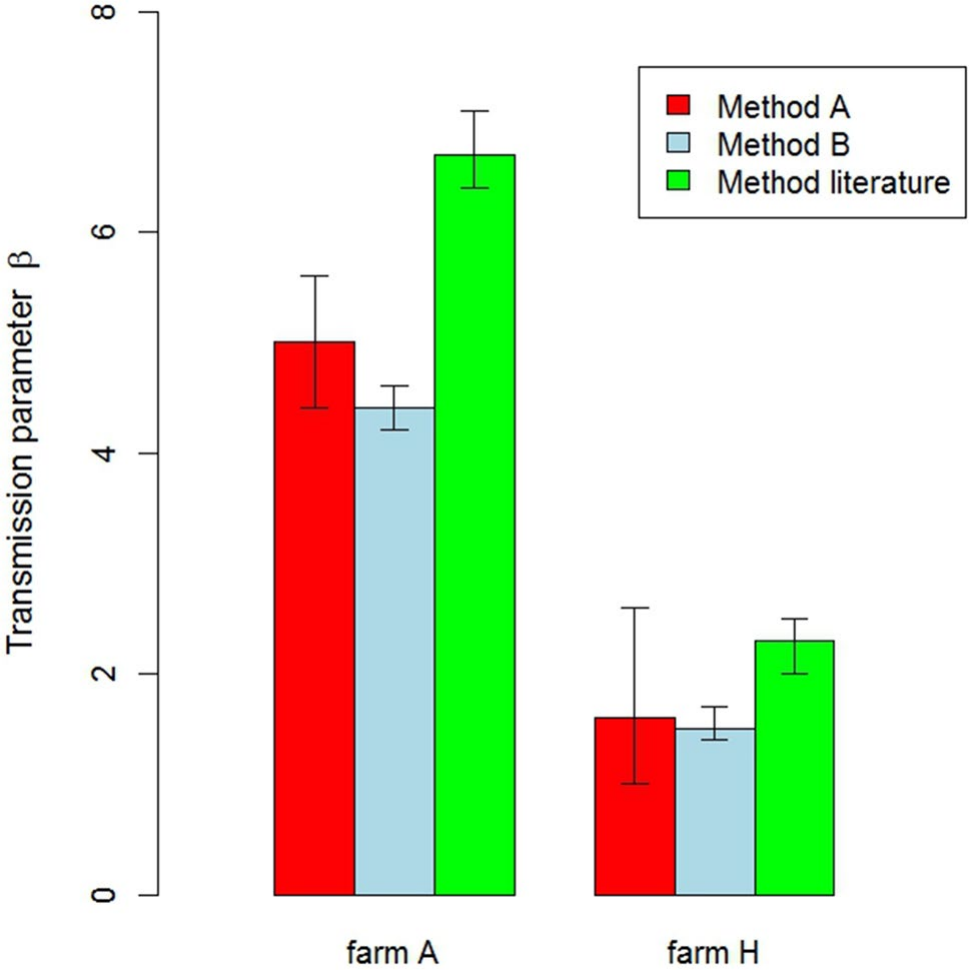
The maximum likelihood estimates and 95% confidence bounds of transmission parameter $\beta$ on two outbreak farms according to three different estimation methods (see text)**.**

Estimates for $\beta$ could be obtained for 8 out of the 11 outbreak farms (Table 4). Only for two outbreak farms, $\beta$ could be estimated using both method A and B (Fig. 2). For the other 6 farms, $\beta$ could only be estimated using method A. Method B could not be applied to 9 out of the 11 outbreak farms, because the number of time points ${n}_{t}$ for the estimation of parameter $\beta$, that resulted from the back-calculation procedure on the data, was less than 2 (Table 2) and did not satisfy our minimum criteria for obtaining accurate $\beta$ estimates. Method A could not be applied to 3 out of the 11 outbreak farms, because the disease-induced mortality fluctuated strongly in time and could not be described well by an exponential model (${r}_{l}<0.75$) (1 farm) or the likelihood profile was too flat to obtain a maximum likelihood estimate and confidence bounds (2 farms).

**Table 4:** The farm-specific estimates of transmission parameter $\beta$ according to method A (see text) and the corresponding mean times of virus introduction and introduction windows for outbreaks of highly pathogenic avian influenza of subtype H5N8 on poultry farms in the Netherlands in 2014 and 2016.

Farm identifier^a^	Farm type	Type of $\beta$ estimate		Mean time of virus introduction (days)	Virus introduction window (days)
		MLE^b^	95% confidence bounds		
**Chickens 2014**					
A	Layer	5	(4.4–5.6)	14.8	(14.3–15.3)
B	Layer	34.4	(27.3–44.1)	9.8	(9.5–10.2)
C	Layer	4.4	(2.2–11.4)	11.8	(9.8–14.5)
**Chickens 2016**					
F	Layer	8.5	(7.1–10.6)	7.4	(6.9–7.8)
C	Layer	10.9	(6–21.4)	5.9	(5.1–6.9)
**Ducks 2016**					
H	Meat duck	1.6	(1–2.6)	14.5	(12.3–18.1)
I	Meat duck	11.8	(8.3–18.1)	9.5	(8.8–10.2)
E	Meat duck	0.95	(0.3–2.3)	18.8	(12.9–51)

^a^Only farms for which parameter transmission parameter $\beta$ could be estimated are shown.

^b^Maximum likelihood estimate.

The estimated values of $\beta$ for outbreaks within a single year varied considerably, both for chicken farms as well as for duck farms (Table 4). Maximum likelihood estimates of $\beta$ for outbreaks on chicken farms varied from 4.4 to 34.4 in 2014 and from 8.5 to 10.9 in 2016. For ducks, $\beta$ could only be estimated for outbreaks in 2016 and varied from 0.95 to 11.8.

### Time of virus introduction

The mean time of virus introduction for outbreaks within a single outbreak season also varied considerably for both chicken and duck farms (Table 4). For chicken farms, it was higher for outbreaks in 2014 (9.8 to 14.8 days) than outbreaks in 2016 (5.9 to 7.4 days). For duck farms, the mean time of virus introduction for outbreaks in 2016 (9.5 to 18.8 days) was much higher than for chickens in the same year.

In general, the width of introduction windows was higher for outbreak farms with a wider confidence interval for the transmission parameter *β* and a lower maximum likelihood estimate of parameter *β* (Table 4). The width of the introduction window also varied considerably between outbreaks on chicken farms from 0.7 to 4.7 days. The width of the introduction window for the outbreaks on duck farms varied from 1.4 to 5.8 days, but was much higher (38.1 days) for one outbreak in 2016; correspondingly this outbreak had a comparatively very low estimate for the transmission parameter *β*.

### Comparison of methods for the estimation of transmission parameter *β*

**Figure 3:**
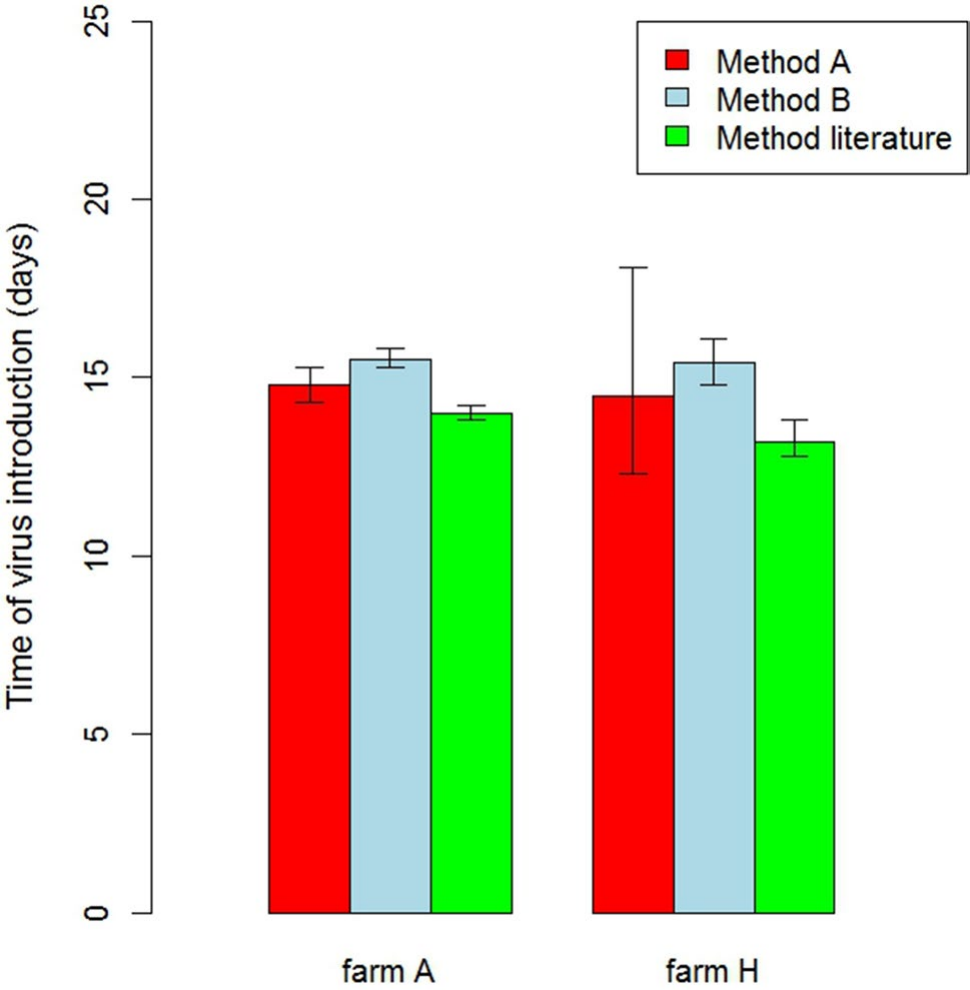
The mean time of virus introduction and the introduction window for two outbreak farms according to three different estimation methods for transmission parameter $\beta$ (see text).

In Figs. 2 and 3 we compare the results of methods A and B for the two outbreak farms where both methods could be applied. The ML estimates for *β* obtained by the two methods were very similar for these outbreaks, but the widths of the 95% confidence interval for *β* were highest for method A. For comparison we also estimated parameter *β* for these two outbreaks using the approximation described by Tiensin et al.^21^ and Bos et al.^20^. This consistently resulted in *β* estimates that were higher than estimates for our methods A and B (Fig. 2). Correspondingly, the predicted mean times of virus introduction were shorter when applying the method described in the literature^20,21^.

### Sensitivity and elasticity analyses

Decreasing the number of data points included in the *β* estimation by one data point could both decrease and increase the estimated values of parameter *β* (see Table 5). The same was true when increasing the number of data points included in the *β* estimation by one data point. As a result, there was also no consistent effect of changes in the number of data points for the *β* estimation on the mean time of virus introduction (Table 5).

**Table 5:** The effect of the number of consecutive days with disease-induced mortality (data points) on the estimated value of transmission parameter $\beta$ and the mean time of virus introduction.

Farm identifier	MLE for transmission rate $\beta$ using method A			Mean time of virus introduction (days to detection)		
	One data point less	Default number of data points	One additional data point	One data point less	Default number of data points	One additional data point
**Chickens 2014**						
A	5.2	5	5	14.6	14.8	14.7
B	34.4	34.4	37	9.8	9.8	9.7
C	–	4.4	4.4	–	11.8	11.9
**Chickens 2016**						
F	5.6	8.5	9.4	8.5	7.4	7.2
C	–	10.9	9.5	–	5.9	6.1
**Ducks 2016**						
H	2.2	1.6	1.8	12.8	14.5	13.9
I	16.5	11.8	10.5	9	9.5	9.7
E	–	0.95	2	–	18.8	13.5

Increasing the length of the latent period increased the estimated value of the transmission parameter *β* for all outbreak farms (see Supplementary Methods & Results S2). The effect of changing the other epidemiological parameters on *β* was smaller and less consistent between outbreak farms. The mean time of virus introduction monotonically increased with increasing length of the latent period and increasing length of the infectious period for animals dying from disease (Supplementary Methods & Results S2).

The elasticity analysis showed that the transmission parameter *β* is most sensitive to changes in the length of the latent period for outbreaks on chicken as well as duck farms (Table 6). The elasticity of the mean time of virus introduction varied much less between epidemiological parameters for outbreaks on both chickens and duck farms. The mean time of virus introduction for outbreaks on duck farms was always least sensitive to changes in the length of the infectious period for recovering individuals.

**Table 6:** The elasticity of transmission rate $\beta$ and the mean time of virus introduction to deviations from the default settings of epidemiological parameters.

Farm identifier	Elasticity of the MLE^a^ for transmission rate ${\varvec{\beta}}$ (using method A) to changes^b^ in the:				Elasticity of the mean time of virus introduction to changes^b^ in the:			
	Latent period	Infectious period (dying animals)	Infectious period (recovering animals)	% dying from disease	Latent period	Infectious period (dying animals)	Infectious period (recovering animals)	% dying from disease
**Chickens 2014**								
A	1.76	− 0.50	–^c^	–^c^	0.17	0.18	–^c^	–^c^
B	1.52	0.18	–^c^	–^c^	0.13	0.24	–^c^	–^c^
C	2.93	− 0.36	–^c^	–^c^	0.14	0.18	–^c^	–^c^
**Chickens 2016**								
F	1.54	− 0.43	–^c^	–^c^	0.25	0.16	–^c^	–^c^
C	0.80	− 0.38	–^c^	–^c^	0.34	0.19	–^c^	–^c^
**Ducks 2016**								
H	0.78	− 0.07	− 0.09	± 0.06^d^	0.12	0.22	± 0.02^d^	− 0.14
I	0.82	0.50	0.00	− 0.34	0.19	0.17	0.00	0.04
E	0.47	0.00	− 0.08	− 0.05	0.13	0.15	− 0.03	− 0.14

^a^Maximum likelihood estimate.

^b^See Supplementary Methods & Results S2 for an overview of the deviations of epidemiological parameters from their default settings.

^c^All infected chickens were assumed to die from disease.

^d^The estimated value of the transmission rate $\beta$ or the mean time of virus introduction did not consistently increase or decrease when the value of the epidemiological parameter was increased.

## Discussion

We developed a stepwise approach for estimating the introduction window of HPAI virus into poultry farms from disease-induced mortality data. A novel aspect of our approach is the focus on the accurate estimation of the farm-specific transmission rate of HPAI virus within poultry flocks, which makes it possible to account for differences in the transmission rate between farm types due to management practices and the design of farm houses. For this purpose, we developed two methods for the estimation of the transmission rate that can be applied to outbreaks in the initial stochastic growth phase (method B) as well as in later outbreak phases (methods A and B). Application to simulated outbreaks with a known transmission rate showed that both methods A and B provided much more accurate estimates than the method most often used in the literature. To demonstrate our approach for estimating the virus introduction window, we applied it to outbreaks of HPAI virus subtype H5N8 in duck and chicken farms in the Netherlands during the years 2014 and 2016.

We explored two different methods for estimating the transmission rate of HPAI virus within flocks. Method A estimates the transmission rate directly from the disease-induced mortality data. In contrast to method B, it assumes a continuous exponential increase in the number of infectious individuals during the initial phase of an outbreak. The fact that it could be successfully applied to 8 out of the 11 outbreaks shows that most outbreaks had already entered the exponential or a later outbreak phase when detected. The exception was the outbreak on one duck farm (farm E in 2014) for which the daily mortality fluctuated strongly in time shortly prior to detection. One must realize that we rely on self-reported mortality data recorded by poultry farmers and that the accuracy of these records will vary between poultry farmers during a clinical episode with high potential economic and emotional impact. The two remaining farms to which method A could not be successfully applied did show exponential growth, but the likelihood functions were too flat to reliably estimate the transmission rate. This is because method A requires the estimation of a second model parameter in addition to the transmission rate.

The second method (B) could only be applied successfully to 2 out of the 11 outbreak farms. This is because this method does not estimate the transmission rate directly from the disease-induced mortality data, but indirectly from the back-calculated number of infectious individuals in time and the number of new cases produced by these individuals during one-day time steps. The number of time steps for which the back-calculation procedure can predict both the number of infectious individuals and the number of new cases depends on two factors: the number of days for which mortality was assumed to be disease-induced and the length of the latent period. This is because the back-calculation procedure first predicts the daily number of new cases from the mortality data and then adds the latent period to predict when these new cases become infectious. For most of the H5N8 outbreaks analysed in this study, disease-induced mortality data was scarce (because poultry farmers reacted fast on a suspicious disease situation). In combination with a latent period of 1 or 2 days, the back-calculation procedure could not provide enough time steps for these outbreaks with information on both the number of infectious individuals and the number of new cases in order to estimate the transmission rate of HPAI virus. The studies in which this back-calculation procedure was introduced for estimating the transmission rate of HPAI did not estimate farm-specific transmission rates, but instead used the back-calculated information from all farms to estimate a farm-independent, i.e. average transmission rate^20,21^. Method B therefore seems better suited for studies at the population rather than at the individual farm level.

For the two outbreaks in this study where both methods A and B could be applied, the estimated transmission rates were very similar, but the confidence intervals were smaller for method B than method A. This is probably because method A involves the estimation of an additional model parameter from the mortality data.

Most of the outbreaks for which disease-induced mortality data was scarce involved poultry flocks that were detected after discovery of the primary case flock in the Netherlands in 2014 and 2016. The three outbreak farms with 4 to 7 days of data on virus-induced mortality were either primary case herds or the first infected farm of a certain type. This scarcity of data on disease-induced mortality may therefore be explained by a higher level of alertness following detection of the primary cases.

To our knowledge, this study is the first to publish estimates of the transmission rate of HPAI H5N8 from field data for domestic chickens as well as domestic ducks. Estimates of the transmission rate of H5N8 for chickens in 2014 and 2016 varied from 4.4 to 34.4 (median 8.5). Published transmission rates of HPAI in domestic chickens for other HPAI subtypes (H5N1, H5N2 and H7N7) varied from 0.73 to 33 in transmission experiments^1,3,27–30^ and from 0.66 to 19.9 in the field^20,21^. It can be concluded that the farm-specific estimates of the transmission rate of HPAI H5N8 virus in domestic chickens are close to or within the range of transmission rates found for other HPAI subtypes. For domestic ducks, the transmission rate was estimated to be 1.22 for HPAI H5N8 virus and 1.6 for HPAI H5N1 virus in transmission experiments^31^. Estimates of the transmission rate in our study were very similar for two out of the three outbreaks on duck farms (0.9–1.6), but much higher for the remaining farm (11.8). It should be noted that the testing of methods A and B using simulated outbreaks with a known transmission rate showed that they may slightly underestimate the value of the transmission rate, but were much more accurate than the method described in the literature^20,21^ above to estimate the transmission rate from field data.

The sensitivity analysis showed that the estimated transmission rates were most sensitive to changes in the latent period. An increase of the latent period by 2 days could increase the transmission rate up to 14.1 times. This sensitivity to changes in the latent period was also observed in other studies^20,24,30^ and is probably caused by the effect of the latent period on the time interval from the moment an individual becomes infected until the first secondary case (generation time). Assuming that the length of the infectious period and the transmission rate remain constant, an increase in the latent period will decrease the growth rate of the number of infectious individuals, since the same amount of secondary infections is produced over a longer period. To achieve a good fit to the observed growth of the number of disease-induced deaths (and therefore infectious individuals), the longer latent period will be compensated for by increasing the value of the transmission rate.

As mentioned above, the estimated transmission rate could vary considerably between farms of the same type that were infected in the same avian influenza season, e.g. from 4.4 to 34.4 for outbreaks on layer farms in 2014 and from 1.22 to 11.8 on meat duck farms in 2016. Several studies have explored the effect of flock age and flock size on the transmission rate of HPAI in poultry flocks. Flock size did not significantly influence the transmission rate of HPAI virus in chicken and turkey farms^20,32^. Flock age did significantly influence the transmission rate of HPAI virus in chicken flocks with lower transmission in older flocks^20^. This age-effect was however not found for turkey flocks^32^. Differences in the density of chickens may also cause variation in the transmission rate, but the birds on all farms included in our analysis were housed indoors and densities in these houses are approximately similar in the Netherlands. In addition, we assumed frequency-dependent transmission in our SEIR model framework with a fixed contact rate independent of the bird density. Finally, the presence of co-morbidities due to other diseases may also cause variation in the within-flock transmission rate. The number of outbreaks included in this study was too small to determine the effect of the different explanatory variables on the transmission rate.

In case of an HPAI outbreak in the Netherlands, the time window for back-tracing contacts will be determined based on epidemiological and other relevant factors (e.g. meteorological)^33^. Our study shows that the most likely time of HPAI introduction was ≤ 14.8 days prior to disease detection for outbreaks on chicken farms and ≤ 18.8 days prior to disease detection for outbreaks on duck farms. The sensitivity analysis showed that these estimates are robust to changes in the values of epidemiological parameters (Supplementary Methods & Results S2). Using conservative estimates of the transmission rate (lower bound of the 95% confidence intervals) also did not increase the time of virus introduction very much for outbreaks on chicken farms and most duck farms as well (Table 4). However, there is one exception. The virus introduction time increased to 51 days prior to detection on one meat duck farm (farm E 2016), when using the conservative estimate of the transmission rate. Our analysis suggests that in most cases a back-tracing window of approximately 3 weeks would be sufficient to capture the period during which a chicken or duck farm is infectious. This information can be used to improve both the efficiency and efficacy of contact-tracing. The uncertainty in the estimation of the introduction window can be reduced by the more accurate collection of mortality data by farmers. It should be noted that all chicken farms for which the transmission rate could be estimated in this study were layers. In addition, it should be noted that the values of epidemiological parameters for future HPAI strains may be different from the H5N8 strains in this study, especially for ducks.

The approach that we developed in this study for the estimation of the introduction window of HPAI onto poultry farms can account for differences in the epidemiology between HPAI subtypes and poultry species by adjusting the values of epidemiological parameters in the SEIR framework. The approach can be extended to other livestock diseases by including other clinical signs than mortality and by including seroprevalence data, as long as these can be predicted using the SEIR framework.

A limitation of our modelling framework is that it does not account for environmental transmission of HPAI. Infectious birds will shed virus contaminating e.g. bedding material and drinking water and it is known that highly pathogenic avian influenza can survive for longer periods of time in the environment^34^. Some models have been developed that account for both direct and environmental transmission in flocks^35,36^, but the relative contribution of both transmission pathways was not estimated in these studies by fitting the model to data. Experimental studies that quantify both direct and environmental transmission of HPAI in poultry flocks have not yet been conducted to our knowledge. To separate the direct and environmental transmission pathways in a flock, we would therefore need data on disease-induced mortality as well as the viral load in the environment. Data on the environmental HPAI load are not collected by farmers. Therefore, we were unable to separate direct and environmental transmission in our models.

A possible improvement to our method would be to determine both the transmission rate and the virus introduction window by fitting the stochastic SEIR model directly to the disease-induced mortality data. This would reduce the number of steps in our method, since the farm-specific transmission rate does not have to be estimated in a separate step. Methods to fit stochastic simulation models to data include both frequentist approaches such as iterated filtering^37^ as well as Bayesian approaches such as Approximate Bayesian Computation^38^, Data Augmentation Markov-Chain Monte Carlo^39^, and particle Markov-Chain Monte Carlo^40^.

A possible disadvantage of fitting a stochastic model directly to the mortality data is the computational burden, which is likely to be higher than when fitting a deterministic model to data. Since the back-tracing of sources during an epidemic requires fast methods to estimate the virus introduction window, the computational burden is important when developing a method for estimating virus introduction windows. We aim to explore the possibility and usefulness of estimating the transmission rate as well as virus introduction window in one step by fitting the stochastic SEIR model directly to the data in future studies.

To conclude, we developed an approach for estimating the introduction window of HPAI virus into individual poultry farms using farm-specific estimates of the transmission rate. We successfully applied this method to 8 out of 11 outbreaks of HPAI virus subtype H5N8 into chicken and duck farms in the Netherlands in 2014 and 2016. The results of our analysis can be used to improve the efficiency and efficacy of contact-tracing efforts by focusing on the time window that a farm was infectious. To our knowledge this study reports the first estimates of the transmission rate of HPAI virus subtype H5N8 in flocks of domestic chickens and ducks.

## Supplementary information

Supplementary information

## Data Availability

The datasets generated during and/or analyzed during the current study are available from the corresponding author on reasonable request.
