# Supplementary material for: Estimating the introduction time of highly pathogenic avian influenza into poultry flocks
Source: Sci Rep. 2020 Jul 24;10:12388. doi: 10.1038/s41598-020-68623-w (PMC7381656; doi:10.1038/s41598-020-68623-w)
Supplement: Supplementary file 1 — Supplementary information [file 41598_2020_68623_MOESM1_ESM.docx]

**­Supplementary Information**

**Estimating the introduction time of highly pathogenic avian influenza into poultry flocks**

Peter H.F. Hobbelen^1*^, Armin R.W. Elbers^1^, Marleen Werkman^1,#a^, Guus Koch^1^, Francisca C. Velkers^2^, Arjan Stegeman^2^ & Thomas J. Hagenaars^1^

^1^ Wageningen Bioveterinary Research, Houtribweg 39, 8221 RA, Lelystad, the Netherlands

^2^ Department of Farm Animal Health, Faculty of Veterinary Medicine, Utrecht University, Utrecht, the Netherlands

^#a^ Current address: Julius Centre for Health Sciences and Primary Care, University Medical Centre Utrecht, Utrecht, The Netherlands.

**Contents**

- Methods S1: The stochastic SEIR model

* Tables S1 to S3

- Methods S2: The estimation of parameters from the literature

* Tables S4 to S7

- Methods S3: The deterministic SEIR model

- Methods S4: The back-calculation procedure

* Tables S8 to S9

- Methods S5: Correcting the transmission rate for the rapid increase in the

number of infectious birds

* Fig. S1

- Methods and Results S1: Model testing

* Tables S10 to S11

* Fig. S2

- Methods and Results S2: Sensitivity analysis

* Tables S12 to S15

- Results S1: The percentage of simulated epidemics dying out in the initial stochastic phase

* Table S16

- References

**Methods S1: The stochastic SEIR model**

The stochastic version of the general SEIR model divides the animal population into susceptible ($S$), latent ($E$), infectious ($I_{R}$ and $I_{D}$), recovered ($R$) and dead ($D$) stages. At the end of the latent stage, a fraction of animals ($f_{D}$) moves into the infectious stage for animals dying from disease and the remainder moves into the infectious stage for surviving animals and will enter the recovered stage. Animals in all disease stages may also die from natural causes at a constant rate $\mu$, although this background mortality is assumed to be negligible compared to disease-induced mortality when estimating the virus introduction time.

The lengths of the latent and infectious stages are gamma distributed. A gamma distribution is defined by a shape parameter $k$ and a scale parameter $\theta$. The mean of the distribution equals $k\theta$ and is set to the estimated length of a certain disease stage ($l_{stage}$). Shape parameter $k$ was determined from literature data (see Methods S2) and set to 20 for all latent and infectious stages. The value of the scale parameter $\theta$ then equals $l_{stage}/k$. This distribution can be built into simulation models by dividing a certain disease stage into $k$ exponentially distributed substages each with a mean length of $l_{stage}/k$ ^1^.

The number of individuals transiting from a given stage to the next during a small time step $dt$ is randomly drawn from a binomial distribution $B(n,p)$ with parameter n representing the total number of individuals in that stage and p representing the transition probability during the small time step. In the model simulations, we set time-step $dt$ to 0.01 day, which is small compared to the lengths of the different disease stages (see Methods S2 below).

The definition of state variables and parameters in the stochastic simulation model are given in Tables S1 and S2 below. The infection probability and transition probabilities between sub-stages during a small time step $dt$ are given in Table S3 below.

**Table S1.** The definition of the state variables in the stochastic and deterministic SEIR model (see main text).

| **State variable** | **Definition** |
| --- | --- |
| $S$ | Number of susceptible individuals |
| $E_{i}$ with $i \in\left[ 1,k \right]$ | Number of individuals in substage $i$ of the latent stage |
| $I_{R,i}$ with $i \in\left[ 1,k \right]$ | Number of individuals in substage $i$ of the infectious stage for recovering individuals |
| $I_{R}$ | The total number of individuals in the infectious stage for recovering individuals |
| $I_{D,i}$ with $i \in\left[ 1,k \right]$ | Number of individuals in substage $i$ of the infectious stage for recovering animals |
| $I_{D}$ | The total number of individuals in the infectious stage for animals dying from disease |
| $R$ | The cumulative number of recovered individuals |
| $D_{B}$ | The cumulative number of individuals having died from natural causes (background mortality) |
| $D_{D}$ | The cumulative number of individuals having died from disease |
| $N$ | The total number of alive individuals (population size) |

**Table S2.** The definition and dimensions of parameters in the stochastic and deterministic SEIR model (see main text).

| **Parameter** | **Definition** | **Dimension** |
| --- | --- | --- |
| $\beta$ | Transmission rate | 1/Day |
| $l_{E}$ | Length of the latent period | Day |
| $\mu$ | Background mortality rate | 1/Day |
| $f_{D}$ | Fraction of infected individuals dying from disease | - (fraction) |
| $l_{IR}$ | Length of the infectious period for recovering individuals | Day |
| $l_{ID}$ | Length of the infectious period for dying individuals | Day |
| $k$ | The shape parameter of the gamma distribution for the lengths of the latent and infectious stages | - (integer number) |

**Table S3.** The transition probabilities between different disease (sub)stages during a small time step $dt$ used in the fixed-timestep approximation of the stochastic SEIR model

| **Transition between:** | **Probability during time interval** $\boldsymbol{dt}$ |
| --- | --- |
| Any stage with living animals to the stage containing animals having died from natural mortality | $1-e^{-\mu dt}$ |
| Susceptible to first exposed sub-stage ($S\to E_{1}$) | $1-e^{-\frac{\beta I}{N}dt}$ |
| Subsequent exposed sub-stages ($E_{i}\to E_{i+1}$) | $1-e^{-\frac{k}{l_{E}}dt}$ |
| Last exposed sub-stage to first infectious sub-stages ($E_{k}\to I_{R,1}$ and $E_{k}\to I_{D,1}$) | $(1-f_{D})\left( 1-e^{-\frac{k}{l_{E}}dt} \right)$ (recovering)  $f_{D}\left( 1-e^{-\frac{k}{l_{E}}dt} \right)$ (dying) |
| Subsequent infectious sub-stages  ($I_{R,i}\to I_{R,i+1}$ and $I_{D,i}\to I_{D,i+1}$) | $1-e^{-\frac{k}{l_{IR}}dt}$ (recovering)  $1-e^{-\frac{k}{l_{ID}}dt}$(dying) |
| Last infectious sub-stages to the recovered or dead stage ($I_{R,k}\to R$ and $I_{D,k}\to D$) | $1-e^{-\frac{k}{l_{IR}}dt}$ (recovering)  $1-e^{-\frac{k}{l_{ID}}dt}$ (dying) |

**Methods S2: The estimation of parameters from the literature**

**The estimation of epidemiological parameters for HPAI H5N8 outbreaks on poultry farms in 2014**

We conducted a literature review to estimate the epidemiological parameters for HPAI subtype H5N8 outbreaks in chickens and ducks in 2014. The databases, search terms and selection criteria for the different stages of this literature review are shown in Table S4 below. This table also shows the type of information to be extracted from the literature review. The extracted information on contact experiments with chickens showed that the mortality among inoculated individuals was usually much higher than the mortality among contact chickens. The steep increase in the daily number of dead chickens that was observed on outbreak farms suggests that the percentage mortality due to disease is very high. For the estimation of model parameters for chickens, we therefore only included data from experiments with 100% disease-induced mortality. This finally resulted in a selection of 11 papers for chickens. Tables S5 and S6 below show the information that was extracted from these 11 studies on chickens and the only study with disease-induced mortality that we could find on ducks.

*The parameter values for chickens in 2014*

Latent period: In most studies, the first cloacal/oropharyngeal swabs were taken at two or more days post infection (dpi) and were positive for the virus. In only two studies, virus shedding was already measured at 1 dpi. One of these studies showed that shedding already occurred on 1 dpi, while the other did not find evidence of virus shedding until 2 dpi. Since the latter study involved a larger number of animals and exposure to different types of the H5N8 virus, we decided to set the default length of the latent period for chickens in 2014 to two days. The possibility of a shorter latent period was explored in the sensitivity analysis (see the main text and Results S2).

Infectious period: The mean time to death (MTD) was reported for all 11 studies on chickens with an overall mean of 4.5 days. Deducting the length of the latent period (see above) from this mean time to death gives an estimate of 2.5 days for the infectious period of chickens in 2014.

Percentage dying from disease: as mentioned above, we assumed 100% disease-induced mortality.

The shape parameter of the gamma distribution: The mean and variance of the gamma distribution with shape parameter $k$ and scale parameter $\theta$ equal $k\theta$ and $k\theta^{2}$. Rearranging the expression for the mean gives $k={mean}/\theta$. Substitution into the equation for the variance subsequently gives $k={{mean}^{2}}/{var}$. The shape parameter of the gamma distribution for the latent and infectious period for chickens was therefore derived from data on mean and variance of the time to death for inoculated individuals. This type of data was reported for 6 out of the 11 studies on chickens. We estimated the value of the shape parameter $k$ for chickens in 2014 as the overall mean for the different studies, giving $k=20$. The mean time to death is the sum of the latent and infectious periods. In absence of individual level data for the length of these specific disease stages, we assumed that the value of the shape parameter for the latent and infectious disease stages was the same as the one estimated for the mean time to death.

*The parameter values for ducks in 2014*

There was only one study in which infected ducks died from disease ^2^. This study consisted of the same contact experiment repeated for three different H5N8 strains. Disease-induced mortality occurred among inoculated ducks in two experiments, but was nil for the contact ducks. There was no disease-induced mortality in the third experiment. We therefore estimated the lengths of the latent and infectious periods from the data on inoculated ducks in the two experiments with disease-induced mortality.

Latent period: In both these experiments, virus shedding already occurred on 1 dpi, so we set the default length of the latent period for ducks in 2014 to one day.

Infectious period: The mean time to death in these experiments amounted to 8 and 11 days, respectively. Deducting the one-day latent period results in an average infectious period for dying animals of 8.5 days. The shedding periods of surviving animals in these two experiments were 7 and 10 days, respectively, resulting in an average infectious period for recovering animals of 8.5 days.

Percentage dying from disease: The percentage of animals dying from disease was 20 in both experiments and this was therefore used as default, too.

The shape parameter of the gamma distribution: In the absence of data, we set the values of the shape parameters for the distributions of the latent and infectious periods to the values estimated for chickens in 2014.

**The estimation of epidemiological parameters for HPAI H5N8 outbreaks on poultry farms in 2016**

The epidemiological parameters for HPAI subtype H5N8 outbreaks in chickens and ducks in 2016 were derived from recently published transmission experiments ^3^.

*The parameter values for chickens in 2016*

The parameter values for chickens were derived from a transmission experiment in which 10 sentinel chickens were exposed to inoculated ducks at 2 dpi. Virus shedding by the sentinel chickens was measured daily until all sentinel chickens had died.

Latent period: The first day of shedding varied from 2 to 5 days post contact (dpc). Since the time of infection of the sentinel chickens is unknown, it is not possible to accurately determine the length of the latent period. We assumed the length of the latent period to be 1 day, because virus shedding was first detected at 2 dpc.

Infectious period: Death occurred at 3 to 7 dpc. The length of the infectious period was estimated as the number of days between the start of virus shedding and death. A number of chickens died on the same day as virus shedding was detected for the first time. These were assumed to have an infectious period of 0.5 days. This gave a mean length for the infectious period of 1.1 days.

Percentage dying from disease: This was set to 100%, since all sentinel chickens died.

The shape parameter of the gamma distribution: In the absence of data, we set the values of the shape parameters for the distributions of the latent and infectious periods to the values estimated for chickens in 2014.

*The parameter values for ducks in 2016*

The parameter values for ducks were derived from a transmission experiment in which 10 ducks were inoculated and two sentinel ducks were exposed to the inoculated ducks at 1 dpi. Because of the small number of sentinel ducks, we only used the data on inoculated ducks for parameter estimation. Virus shedding by the inoculated ducks was measured daily until 7 dpi and then at 10 and 16 dpi. All ducks shed virus.

Latent period: Virus shedding was detected at 1 dpi for all ducks and we assumed the length of the latent period to be 1 day.

Infectious period: Two out of the 10 inoculated ducks died at 4 and 5 dpi. Subtracting the latent period gives a mean infectious period for dying animals of 3.5 days. All surviving chickens shed virus at 7 dpi but not at the next sampling time at 10 dpi. We assumed that virus shedding ended at 7 dpi. Subtracting the latent period gives a mean infectious period for surviving animals of 6 days.

Percentage dying from disease: This was set to 20%, since 2 out of 10 ducks died.

The shape parameter of the gamma distribution: In the absence of data, we set the values of the shape parameters for the distributions of the latent and infectious periods to the values estimated for chickens in 2014.

**Table S4.** The databases, search terms, selection criteria and the type of information to be extracted for the literature review on epidemiological parameters of HPAI subtype H5N8 in chickens and ducks for the 2014 outbreaks.

| **Stage of the literature review** | **Selection criteria/extracted information** | **Number of publications** |
| --- | --- | --- |
| Databases | Scopus, Opus and Pubmed | - |
| Search terms | ([Title/Abstract]: H5N8) AND  ([All fields]: infectious OR latent OR epidemiology OR transmission) | 158 |
| Abstract screening | (Farm type: poultry) AND  (Virus: H5N8) AND  (Type of study: transmission experiment OR challenge experiment OR outbreak analysis to study epidemiological parameters (life-history)) | 21 |
| Full text screening | (100% mortality due to disease for chickens; any disease-induced mortality for ducks) AND  (The mean time to death OR the virus shedding period) | 11 studies for chickens and 1 study for ducks |
| Extracted information | - Type of study (Challenge/Contact)  - Poultry species  - Age at infection  - Number of animals  - Virus name  - Virus clade  - Exposure dose  - Mean time to death  - Percentage disease-induced mortality  - First day of virus shedding post infection  - Day post infection at which the first cloacal or oropharyngeal swab was taken  - Last day of virus shedding | - |

**Table S5.** The literature data that was used to estimate the length of the latent and infectious period of chickens infected by highly pathogenic avian influenza of subtype H5N8 on poultry farms in the Netherlands in 2014. Only data from experiments with inoculated non-vaccinated chickens were used with 100% disease-induced mortality.

| **Virus name** | **Type of chicken^a^** | **Number of exposed**  **Animals** | **Exposure**  **Dose (EID_50_)^b^** | **Mean time to death (days)** | **First swabs taken (dpi)^c,d^** | **First day that virus was detected in swabs (dpi)** | **Source** |
| --- | --- | --- | --- | --- | --- | --- | --- |
| MDk/W452 H5N8 | commercial layer | 2 | 10^4^ | 4.5 | -^e^ | -^e^ | ^4^ |
| MDk/W452 H5N8 | commercial layer | 2 | 10^5^ | 4 | -^e^ | -^e^ | ^4^ |
| MDk/W452 H5N8 | SPF white leghorn | 4 | 10^3^ | 6 | -^e^ | -^e^ | ^4^ |
| MDk/W452 H5N8 | SPF white leghorn | 4 | 10^4^ | 4 | -^e^ | -^e^ | ^4^ |
| MDk/W452 H5N8 | SPF white leghorn | 4 | 10^5^ | 4.5 | -^e^ | -^e^ | ^4^ |
| A/gyrfalcon/Washington/40188-6/2014 (H5N8) | SPF | 5 | 10^6.3^ | 4.1 | 1 | 1 | ^5^ |
| A/chicken/Kumamoto/1-7/2014 (H5N8) | SPF | 3 | 10^7.8^ | 4.7 | 2 | 2 | ^6^ |
| A/Gyrfalcon/Washington/2014 (H5N8) | SPF | 10 | 10^6^ | 3.7 | 2 | 2 | ^7^ |
| A/MD/W452/14 (H5N8) | SPF white leghorn | 18 | 10^4.5^ | 4 | 3 | 3 | ^8^ |
| A/broiler duck/Korea/Buan2/2014 (H5N8) | Korea native chicken | 5 | 10^7.5^ | 5 | -^e^ | -^e^ | ^9^ |
| A/broiler duck/Korea/Buan2/2014 (H5N8) | Korea native chicken | 5 | 10^8.5^ | 4.4 | -^e^ | -^e^ | ^9^ |
| A/broiler duck/Korea/Buan2/2014 (H5N8) | SPF white leghorn | 5 | 10^6.5^ | 3.2 | -^e^ | -^e^ | ^9^ |
| A/Baikal teal/Korea/K14-E016/2014 (H5N8) | commercial layer | 6 | 10^6^ | 4.8 | 2 | 2 | ^9^ |
| A/breeder duck/Korea/Gochang1/2014 (H5N8) | SPF | 8 | 10^6.5^ | 2.5 | 1 | 2 | ^10^ |
| A/broiler duck/Korea/Buan2/2014 (H5N8) | SPF | 8 | 10^6.5^ | 4.5 | 1 | 2 | ^10^ |
| A/broiler duck/Korea/Buan2/2014 (H5N8) | SPF | 3 | 10^6.5^ | 4.3 | 1 | 2 | ^10^ |
| A/Turkey/Germany-MV/R2472/2014 (H5N8) | SPF white leghorn | 10 | 10^6^ | 3 | 2 | 2 | ^11^ |
| A/chicken/Miyazaki/7/2014 (H5N8) | SPF white leghorn | 5 | 10^6^ | 5 | 2 | 2 | ^12^ |
| A/chicken/Miyazaki/7/2014 (H5N8) | SPF white leghorn | 5 | 10^7^ | 3.8 | 2 | 2 | ^12^ |
| A/duck/Chiba/26-372-48/2014 (H5N8) clade | SPF white leghorn | 4 | 10^4^ | 7 | 2 | 2 | ^12^ |
| A/duck/Chiba/26-372-48/2014 (H5N8) clade | SPF white leghorn | 4 | 10^5^ | 8 | 2 | 2 | ^12^ |
| A/duck/Chiba/26-372-48/2014 (H5N8) clade | SPF white leghorn | 4 | 10^5^ | 4 | 2 | 2 | ^12^ |
| A/duck/Chiba/26-372-48/2014 (H5N8) clade | SPF white leghorn | 4 | 10^7^ | 4 | 2 | 2 | ^12^ |
| A/duck/Liaoning/S1001/14 (H5N8) | SPF white leghorn | 5 | 10^5^ | 4 | 3 | 3 | ^13^ |

^a^) SPF = Susceptible Pathogen Free chicken

^b^) EID_50_ = 50% egg infective dose

^c^) dpi = days post infection

^d^) Swabs were either taken from the cloaca or the oropharynx

^e^) Not determined or reported

**Table S6.** The literature data that was used to estimate the length of the latent and infectious period of ducks infected by highly pathogenic avian influenza of subtype H5N8 on poultry farms in the Netherlands in 2014. Only data from experiments with inoculated non-vaccinated ducks were used in which disease-induced mortality occurred.

| **Virus name** | **Type of duck** | **Number of**  **exposed**  **animals** | **Exposure**  **dose (EID_50_)^a^** | **Percentage mortality due to disease (%)** | **Mean time to death (days)** | **First swabs taken (dpi)^b,c^** | **First day that virus was detected in swabs (dpi)** | **Shedding period for surviving animals (days)** | **Source** |
| --- | --- | --- | --- | --- | --- | --- | --- | --- | --- |
| A/Baikal teal/Korea/  Donglim3/2014 (H5N8) | Pekin ducks,  commercially  available | 5 | 10^6.5^ | 20 | 11 | 1 | 1 | 10 | ^2^ |
| A/breeder duck/Korea/  Gochang1/2014 (H5N8) | Pekin ducks,  commercially  available | 3 | 10^6.5^ | 20 | 8 | 1 | 1 | 7 | ^2^ |

^a^) EID_50_ = 50% egg infective dose

^b^) dpi = days post infection

^c^) Swabs were either taken from the cloaca or the oropharynx

**Table S7.** The derivation of the shape parameter of the gamma distribution describing the time to death for chickens after infection with avian influenza of subtype H5N8 in chickens from literature data.

| **Individual animal** | **Literature source** | | | | | |
| --- | --- | --- | --- | --- | --- | --- |
|  | ^7^ | ^8^ | ^10^ | ^10^ | ^11^ | ^12^ |
| Individual 1 | 3 | 3 | 2 | 2 | 2 | 3 |
| Individual 2 | 3 | 3 | 2 | 3 | 2 | 3 |
| Individual 3 | 3 | 3 | 2 | 3 | 3 | 4 |
| Individual 4 | 3 | 3 | 2 | 4 | 3 | 5 |
| Individual 5 | 4 | 3 | 2 | 4 | 3 | 10^a^ |
| Individual 6 | 4 | 3 | 2 | 4 | 3 |  |
| Individual 7 | 4 | 3 | 3 | 5 | 3 |  |
| Individual 8 | 4 | 3 | 3 | 11^a^ | 3 |  |
| Individual 9 | 4 | 3 |  |  | 4 |  |
| Individual 10 | 5 | 5 |  |  | 4 |  |
| Individual 11 |  | 5 |  |  |  |  |
| Individual 12 |  | 5 |  |  |  |  |
| Individual 13 |  | 5 |  |  |  |  |
| Individual 14 |  | 5 |  |  |  |  |
| Individual 15 |  | 5 |  |  |  |  |
| Individual 16 |  | 5 |  |  |  |  |
| Individual 17 |  | 5 |  |  |  |  |
| Individual 18 |  | 5 |  |  |  |  |
| Total number of individuals | 10 | 18 | 8 | 8 | 10 | 5 |
| Mean ($k\theta$) | 3.7 | 4 | 2.3 | 3.6 | 3 | 3.8 |
| Variance ($k\theta^{2}$) | 0.46 | 1.1 | 0.21 | 0.95 | 0.44 | 0.92 |
| Scale parameter of the gamma distribution ($\theta$) | 0.12 | 0.26 | 0.1 | 0.27 | 0.15 | 0.24 |
| Shape parameter of the gamma distribution ($k$) | 30.1 | 15.1 | 23.6 | 13.4 | 20.3 | 15.3 |

^a^) These values were considered to be outliers and not taken into account for the calculation of the shape parameter of the gamma distribution for the time to death.

**Methods S3: The deterministic SEIR model**

Method A estimates the value of transmission parameter $\beta$ by fitting an ordinary differential equation (ODE) model to the data on the daily disease-induced mortality using a maximum likelihood approach (see main text). The structure of this ODE-model for ducks and chickens is the same as the structure of the stochastic simulation model described in the Methods S1. The definition of state variables and parameters are given in Tables S1 and S2 in the Methods S1.

Like the stochastic model, the sojourn time of individuals in the exposed and infectious stages was assumed to have a gamma distribution. In order to achieve this, these stages were divided into $k$substages with parameter $k$ denoting the shape parameter of the gamma distribution ^1^. The transition rate between subsequent exposed substages is $\frac{k}{l_{E}}$ with parameter $l_{E}$ representing the mean length of the exposed stage. The transition rates from the last exposed substage to the first infectious stages is $(1-f_{D})\frac{k}{l_{E}}$ for surviving animals and $f_{D}\frac{k}{l_{E}}$ for animals that will die from disease. The transition rate between subsequent infectious substages or from the last infectious substage to the recovered or dead stage is $\frac{k}{l_{I_{\text{D}}}}$ for animals that will die from disease and $\frac{k}{l_{I_{\text{R}}}}$ for recovering animals. Parameters $l_{I_{D}}$ and $l_{I_{R}}$ represent the mean length of the exposed and infectious stages, respectively. Below, the equations for the different stages are given.

*Susceptible stage*

$\frac{dS}{dt}=-\frac{\beta\left( I_{D}+I_{R} \right)}{N_{0}}S-\mu S$ (S1)

*Latent stage (gamma distributed with shape parameter* $k$ *and subscript* $i$ *indicating the substage)*

$\frac{dE_{1}}{dt}=\frac{\beta\left( I_{D}+I_{R} \right)}{N}S-\frac{k}{l_{E}}E_{1}-\mu E_{1}$ (S2)

$\frac{dE_{i}}{dt}=\frac{k}{l_{E}}E_{i-1}-\frac{k}{l_{E}}E_{i}-\mu E_{i}$ for $i \in\left[ 2,k \right]$

*Infectious stage for recovering individuals* (gamma distributed with shape parameter $k$ and subscript $i$ indicating the substage)

$\frac{dI_{R,1}}{dt}={(1-f}_{D})\frac{k}{l_{E}}E_{k}-\frac{k}{l_{IR}}I_{R,1}-\mu I_{R,1}$ (S3)

$\frac{dI_{R,i}}{dt}=\frac{k}{l_{IR}}I_{R,i-1}-\frac{k}{l_{IR}}I_{R,i}-\mu I_{R,i}$ for $i \in\left[ 2,k \right]$

*Infectious stage for individuals dying from disease* (gamma distributed with shape parameter $k$ and subscript $i$ indicating the substage)

$\frac{dI_{D,1}}{dt}=f_{D}\frac{k}{l_{E}}E_{k}-\frac{k}{l_{ID}}I_{D,1}-\mu I_{D,1}$ (S4)

$\frac{dI_{D,i}}{dt}=\frac{k}{l_{ID}}I_{D,i-1}-\frac{k}{l_{ID}}I_{D,i}-\mu I_{D,i}$ for $i \in\left[ 2,k \right]$

*Recovered stage*

$\frac{dR}{dt}=\frac{k}{l_{IR}}I_{R,k}-\mu R$ (S5)

*Dead due to disease stage*

$\frac{dD_{D}}{dt}=\frac{k}{l_{ID}}I_{D,k}$ (S6)

*Dead due to background mortality stage*

$\frac{dD_{B}}{dt}=\mu(S+\sum_{i=1}^{i=k} E_{i}+\sum_{i=1}^{i=k} I_{R,i}+\sum_{i=1}^{i=k} I_{D,i}+R)$ (S7)

We numerically integrated these ordinary differential equations using a method which applies a variable step size in order to balance computational efficiency with a sufficient level of accuracy (function lsoda in R package deSolve).

**Methods S4: The back-calculation procedure**

**Table S8.** The definition of additional parameters and variables that were used in the equations of the back-calculation process of method B (Table S9 below). See Table S2 for the meaning of parameters that were defined earlier.

| **Parameter/Variable** | **Definition** |
| --- | --- |
| $n_{d}$ | The number of days for which mortality was assumed to be disease-induced. |
| $t_{0}$ (set to value 0 by definition) | Point in time at which the individuals were infected that died on the first day for which mortality was assumed to be due to disease. This is assumed to be the start of the outbreak for the purpose of the back-calculation. |
| $t_{d}=t_{0}+n_{d}+l_{E}+l_{ID}-1$ | The last day for which disease-induced mortality was recorded |
| $M(t)$ | The disease-induced mortality on day $t$ |

**Table S9.** The equations used in method B for back-calculating the number of susceptible and infectious individuals and the daily number of new cases from the disease-induced mortality that was observed on farms.

| **Variable** | **Definition** |
| --- | --- |
| $C_{D}\left( t \right)=\left\{ \begin{matrix} M\left( t+l_{E}+l_{ID} \right) & if & {t_{0}}^{a}\leq t\leq t_{0}+n_{d}-1 \\ {NA}^{b} & if & t_{0}+n_{d}\leq t\leq t_{d} \end{matrix} \right.$ | The number of new cases at time $t$ that will die from disease |
| $C_{R}\left( t \right)=\left\{ \begin{matrix} \left( \frac{1-f_{D}}{f_{D}} \right) M(t+l_{E}+l_{ID}) & if & t_{0}\leq t\leq t_{0}+n_{d}-1 \\ NA & if & t_{0}+n_{d}\leq t\leq t_{d} \end{matrix} \right.$ | The number of new cases at time $t$ that will recover |
| $C\left( t \right)=\left\{ \begin{matrix} C_{R}\left( t \right)+C_{D}(t) & if & t_{0}\leq t\leq t_{0}+n_{d}-1 \\ NA & if & t_{0}+n_{d}\leq t\leq t_{d} \end{matrix} \right.$ | The total number of new cases at time $t$ |
| $E\left( t \right)=\left\{ \begin{matrix} \sum_{i=0}^{i=min(l_{E}-1,t)} C(t-i) & if & t_{0}\leq t\leq t_{0}+n_{d}-1 \\ NA & if & t_{0}+n_{d}\leq t\leq t_{d} \end{matrix} \right.$ | The number of individuals in the latent stage at time $t$ |
| $I_{D}\left( t \right)=\left\{ \begin{matrix} 0 & if & t_{0}\leq t\leq t_{0}+l_{E}-1 \\ \sum_{i=l_{E}}^{i=min(l_{E}+l_{ID}-1,t)} f_{D}C\left( t-i \right) & if & t_{0}+l_{E}\leq t\leq t_{0}+l_{E+}n_{d}-1 \\ NA & if & t_{0}+l_{E}+n_{d}\leq t\leq t_{d} \end{matrix} \right.$ | The number of infectious individuals at time $t$ that will eventually die from disease |
| $I_{R}\left( t \right)=\left\{ \begin{matrix} 0 & if & t_{0}\leq t\leq t_{0}+l_{E}-1 \\ \sum_{i=l_{E}}^{i=min(l_{E}+l_{IR}-1,t)} {(1-f}_{D})C\left( t-i \right) & if & t_{0}+l_{E}\leq t\leq t_{0}+l_{E+}n_{d}-1 \\ NA & if & t_{0}+l_{E+}n_{d}\leq t\leq t_{d} \end{matrix} \right.$ | The number of infectious individuals at time $t$ that will eventually recover |
| $I\left( t \right)=\left\{ \begin{matrix} 0 & if & t_{0}\leq t\leq t_{0}+l_{E}-1 \\ I_{R}\left( t \right)+I_{D}\left( t \right) & if & t_{0}+l_{E}\leq t\leq t_{0}+l_{E+}n_{d}-1 \\ NA & if & t_{0}+l_{E+}n_{d}\leq t\leq t_{d} \end{matrix} \right.$ | The total number of infectious individuals at time $t$ |
| ${R\left( t \right)}^{c}=\left\{ \begin{matrix} 0 & if & t_{0}\leq t\leq\min\left( t_{0}+l_{E}+l_{IR}-1,t_{d} \right) \\ \sum_{i=l_{E}+l_{IR}}^{i=t} {(1-f}_{D})C(t-i) & if & t_{0}+l_{E}+l_{IR}\leq t\leq min(t_{0}+l_{E}+l_{IR}+n_{d}-1,t_{d}) \\ NA & if & t_{0}+l_{E}+l_{IR}+n_{d}\leq t\leq t_{d} \end{matrix} \right.$ | The cumulative number of recovered individuals at time $t$ |
| $D\left( t \right)=\left\{ \begin{matrix} 0 & if & t_{0}\leq t\leq t_{d}-n_{d} \\ \sum_{i=0}^{i=t} M(t-i) & if & t_{d}-n_{d}+1\leq t\leq t_{d} \end{matrix} \right.$ | The cumulative number of individuals that died from disease at time $t$ |
| $S\left( t \right)=\left\{ \begin{matrix} N_{0}-E\left( t \right)-I_{R}\left( t \right)-I_{D}\left( t \right)-R\left( t \right)-D\left( t \right) & if & t_{0}\leq t\leq t_{0}+n_{d}-1 \\ NA & if & t_{0}+n_{d}\leq t\leq t_{d} \end{matrix} \right.$ | The number of susceptible individuals at time $t$ |

^a^) By definition, the value of $t_{0}$ is set to 0 (see also Table S8).

^b^) NA indicates that the value of a state variable is unknown for the specified time span.

^c^) Time $t$ can vary from $t_{0}$ to $t_{d}$. As shown in the Table above, the equation for calculating the cumulative number of recovered individuals ($R$) may change when time $t$ moves from $t_{0}$ to $t_{d}$. When the infectious stage for individuals that recover ($l_{IR}$) is long, the lower boundary of the time interval during which a certain equation is valid may become higher than $t_{d}$. In that case, the corresponding equation is not valid and redundant, since $R$ only needs to be calculated for the interval from $t_{0}$ to $t_{d}$.

**Methods S5: Correcting the transmission rate for the rapid increase in the**

**number of infectious birds**

Method B for the estimation of transmission parameter $\beta$ builds on earlier work by Tiensin et al. ^14^ and Bos et al. ^15^. Here the daily infection probability ($p_{inf}$) is approximated assuming a constant force of infection ($\lambda$) as

$p_{inf}(t_{s})=1-e^{-\lambda(t_{s})}$ (S7)

with

$\lambda\left( t_{s} \right)=\frac{\beta I(t_{s})}{N(t_{s})}$. (S8)

In this expression, $I$ represents the total number of infectious individuals and $N$ represents the population size at time $t_{s}$.

Using a constant force of infection may however lead to an overestimation of the transmission parameter $\beta$, because the number of infectious individuals (and therefore the force of infection) may rise very sharply even during a time step of one day. To improve the accuracy of the estimate of parameter $\beta$, we developed an alternative expression for the infection probability, which allows the number of infectious individuals to grow exponentially, in a deterministic fashion, during time step $\Delta t$ instead of remaining constant:

$I\left( t_{s}+\Delta t \right)=I(t_{s})e^{r\Delta t}$ (S9)

with growth rate $r$. Substituting this in the equation for the force of infection (equation S8) gives

$\lambda(t_{s})=\frac{\beta I(t_{s})e^{r\Delta t}}{N(t_{s})}$. (S10)

After integrating over the one-day time step ($\Delta t=1$) we obtain

$p_{inf}(t_{s})=1-e^{-\frac{\beta I(t_{s})(e^{r}-1)}{N(t_{s})r}}$. (S11)

The exponential growth rate of the number of infectious individuals ($r$) can be estimated from the generation time distribution ($k(t)$) and $R_{0}$ according to the equation ^16^

$\frac{1}{R_{0}}=\frac{\int_{0}^{\infty} k(t)e^{-rt}dt}{\int_{0}^{\infty} k(t)dt}$. (S12)

The generation time is the time interval between acquiring an infection and infecting another individual. The reproductive number $R_{0}$ is the number of secondary infections caused by one infectious individual in a completely susceptible population. The generation time for ducks depends on the length of the latent period ($l_{E}$), the lengths of the infectious periods for dying ($l_{ID}$) and surviving animals ($l_{IR}$) and the fraction of infected animals dying from disease $f_{D}$. Given the assumption that all individuals spend exactly the same amount of time in the latent stage and the same amount of time in the infectious stages, the generation time distribution can be expressed as a block function (Fig. S1) with $k\left( t \right)=k_{1}$ at the interval [$l_{E}$,$l_{E}+l_{ID}$] and $k\left( t \right)=k_{2}$ at the interval ($l_{E}+l_{ID}$,$l_{IR}$] with

$k_{1}=\frac{f_{D}}{l_{ID}}+\frac{1-f_{D}}{l_{IR}}$ (S13)

and

$k_{2}=\frac{1-f_{D}}{l_{IR}}$. (S14)

Substituting this block function for $k\left( t \right)$ into equation (S12) gives

$\frac{1}{R_{0}}=\frac{\int_{l_{E}}^{l_{E}+l_{ID}} \left( \frac{f_{D}}{l_{ID}}+\frac{1-f_{D}}{l_{IR}} \right)e^{-rt}dt+\int_{l_{E}+l_{ID}}^{l_{E}+l_{IR}} \left( \frac{1-f_{D}}{l_{IR}} \right)e^{-rt}dt}{\int_{l_{E}}^{l_{E}+l_{ID}} \left( \frac{f_{D}}{l_{ID}}+\frac{1-f_{D}}{l_{IR}} \right)dt+\int_{l_{E}+l_{ID}}^{l_{E}+l_{IR}} \left( \frac{1-f_{D}}{l_{IR}} \right)dt}$. (S15)

The denominator in this equation equals the integral of the distribution for the generation time (probability density function) over the entire time interval and sums to 1. Integrating the numerator then gives

$\frac{1}{R_{0}}=\frac{e^{-rl_{E}}}{r}\left( \frac{f_{D}}{l_{ID}}\left( 1-e^{-rl_{ID}} \right)+\frac{{(1-f}_{D})}{l_{IR}}\left( 1-e^{-rl_{IR}} \right) \right)$. (S16)

For a disease with different infectious periods for surviving and non-surviving individuals, $R_{0}$ equals

$R_{0}=\beta(f_{D}t_{death}+\left( 1-f_{D} \right)t_{inf})$. (S17)

Substitution in equation (S16) and rearranging terms gives the following equation for the exponential growth rate of the number of infectious individuals for ducks

$r=\beta\left( f_{D}t_{death}+(1-f_{D})t_{inf} \right)e^{-rt_{lat}}\left( \frac{f_{D}}{t_{death}}\left( 1-e^{-rt_{death}} \right)+\frac{{(1-f}_{D})}{t_{inf}}\left( 1-e^{-rt_{inf}} \right) \right)$. (S18)

Since all infected chickens are assumed to die from disease, the exponential growth rate for this species can be obtained by substituting $f_{D}=1$into Equation S18, giving

$r=\beta e^{-rt_{lat}}\left( 1-e^{-rt_{death}} \right)$. (S19)

The exponential growth rate $r$ can be solved from this equation by plotting the left and right side of this equation in the same graph and determining the value of $r$ for which both sides have the same value. The value of $r$ can then be substituted into equation (S11) for the infection probability.

**Figure S1.** An example of the distribution for the generation time for ducks using a block function (see text above) with a 1-day-long latent period ($l_{E})$, a 4-day-long infectious period for dying animals ($l_{ID}$), a 6-day-long infectious period ($l_{IR}$) for surviving animals and 20% of infected animals dying from disease ($f_{D}=0.2$).


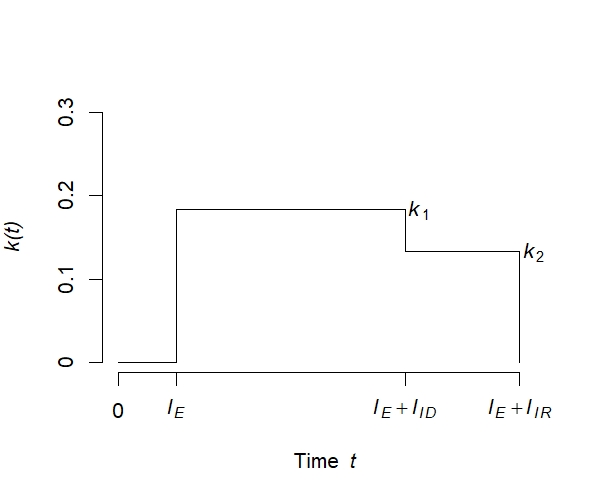


**Methods and Results S1: Model testing**

This supplement shows the estimated values of transmission parameter $\beta$ for simulated outbreaks with transmission rates varying from 2.5 to 10 in steps of 2.5 and the background daily mortality rate set to either 0 or 0.02% for each value of the transmission rate. For each combination of transmission and background mortality rate, we simulated one outbreak using the deterministic version of the general SEIR model (Supplementary Methods S3) and 200 outbreaks with the stochastic version of the general SEIR model (Supplementary Methods S1). The other parameter settings were the same for both models with a 1-day latent period, a 2-day infectious period, 100% mortality and the shape parameter of the gamma-distribution for the latent and infectious periods was set to 20. All simulations started with 1 latent individual in a poultry house with in total 25,000 birds. (Fig. S2).

**Table S10.** The estimated value and 95% confidence bounds of transmission parameter $\beta$ by methods A, B and the literature method (see main text) for simulated outbreaks with the deterministic and stochastic version of the general SEIR model (Methods S1 and S3) without the presence of background mortality.

| **True value transmission rate** | **Estimated value transmission rate** | | | | | |
| --- | --- | --- | --- | --- | --- | --- |
|  | **Deterministic outbreaks** | | | **Stochastic outbreaks** | | |
|  | **MLE** | **CI low** | **CI high** | **mean MLE** | **CI low^a^** | **CI high^a^** |
| *Method A* | | | | | | |
| 2.5 | 2.5 | 2.1 | 2.9 | 2.3 | 2 | 2.6 |
| 5 | 5 | 4.4 | 5.7 | 4.4 | 3.9 | 4.8 |
| 7.5 | 7.5 | 7.1 | 7.9 | 6.5 | 6.1 | 7 |
| 10 | 10 | 9.4 | 10.7 | 8.4 | 7.8 | 9 |
| *Method B* | | | | | | |
| 2.5 | 2.7 | 2.5 | 2.9 | 2.2 | 1.9 | 2.6 |
| 5 | 5.2 | 5 | 5.5 | 4.4 | 4 | 4.8 |
| 7.5 | 7.5 | 7.3 | 7.7 | 6.4 | 5.9 | 7 |
| 10 | 10.4 | 10.1 | 10.7 | 8.2 | 7.7 | 8.9 |
| *Method Literature* | | | | | | |
| 2.5 | 4.3 | 4 | 4.8 | 3.5 | 2.8 | 4.2 |
| 5 | 10.8 | 10.2 | 11.5 | 8.6 | 7.5 | 9.7 |
| 7.5 | 17.9 | 17.4 | 18.5 | 14.4 | 12.9 | 16.4 |
| 10 | 28.1 | 27.1 | 29.1 | 20.4 | 18.6 | 22.7 |

^a^ The 95% confidence interval is derived from the distribution of the maximum likelihood estimates that was obtained by performing 200 simulations with the stochastic version of the general SEIR model.

**Table S11.** The estimated value and 95% confidence bounds of transmission parameter $\beta$ by methods A, B and the literature method (see main text) for simulated outbreaks with the deterministic and stochastic version of the general SEIR model (Methods S1 and S3) with the daily background mortality set to 0.02%.

| **True value transmission rate** | **Estimated value transmission rate** | | | | | |
| --- | --- | --- | --- | --- | --- | --- |
|  | **Deterministic outbreaks** | | | **Stochastic outbreaks** | | |
|  | **MLE** | **CI low** | **CI high** | **mean MLE** | **CI low^a^** | **CI high^a^** |
| *Method A* | | | | | | |
| 2.5 | 2.2 | 1.7 | 2.7 | 2.0 | 1.6 | 2.5 |
| 5 | 4.7 | 4.3 | 5.3 | 4.1 | 3.3 | 4.5 |
| 7.5 | 7.5 | 7.1 | 7.9 | 6.1 | 5 | 6.7 |
| 10 | 9.5 | 8.9 | 10.6 | 8.0 | 6.9 | 8.8 |
| *Method B* | | | | | | |
| 2.5 | 2.3 | 2.1 | 2.6 | 1.9 | 1.4 | 2.4 |
| 5 | 4.8 | 4.6 | 5.1 | 3.9 | 2.9 | 4.5 |
| 7.5 | 7.3 | 7.1 | 7.5 | 5.9 | 4.5 | 6.7 |
| 10 | 9.6 | 9.3 | 9.9 | 7.7 | 6.3 | 8.5 |
| *Method Literature* | | | | | | |
| 2.5 | 3.6 | 3.2 | 4.1 | 2.8 | 2.0 | 3.7 |
| 5 | 9.7 | 9.1 | 10.3 | 7.4 | 4.9 | 8.9 |
| 7.5 | 17.2 | 16.7 | 17.7 | 12.9 | 8.8 | 15.2 |
| 10 | 25.3 | 24.4 | 26.2 | 18.7 | 14.0 | 21.5 |

^a^ The 95% confidence interval is derived from the distribution of the maximum likelihood estimates that was obtained by performing 200 simulations with the stochastic version of the general SEIR model.

**Figure S2.** The daily mortality during the first 10 days of an outbreak on a chicken farm as predicted by the deterministic and stochastic versions of the general SEIR simulation model with transmission rate $\beta$ set to 5 (see above for the other parameter settings) in the absence of background mortality.


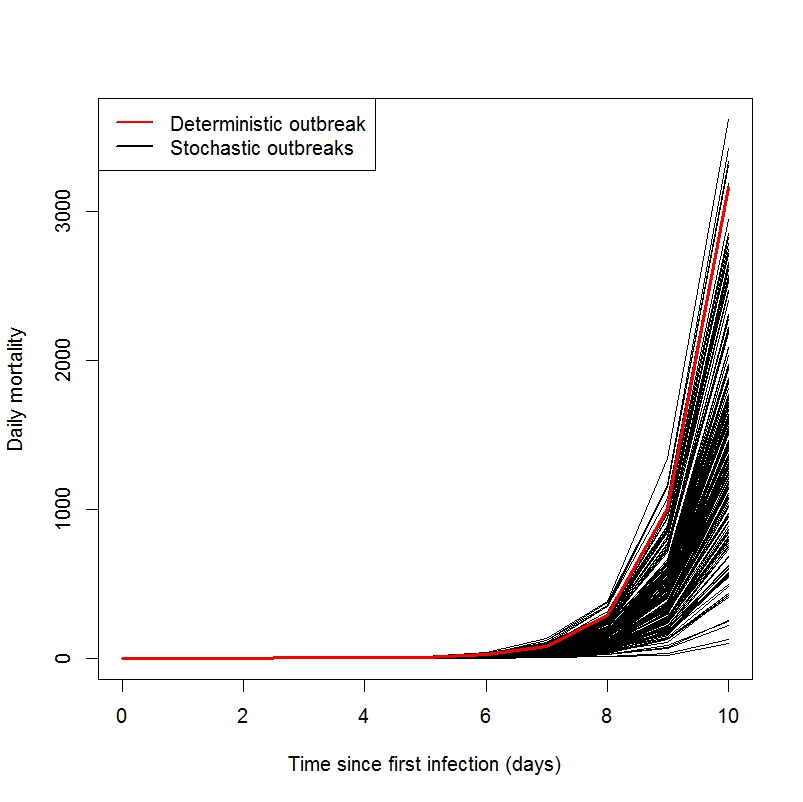


**Methods and Results S3: Sensitivity Analysis**

The tables below show the change in the estimated values of transmission parameter $\beta$ and the mean time of virus introduction due to variation of the following epidemiological parameters:

- the length of the latent period (Table S12)

- the length of the infectious period of individuals dying from disease (Table S13)
- the length of the infectious period for recovering individuals (Table S14)

- the percentage of individuals that die from disease (Table S15)

**Table S12.** The maximum likelihood estimate of transmission parameter $\beta$ as function of the length of the latent period for outbreaks of highly pathogenic avian influenza of subtype H5N8 on Dutch poultry farms in 2014 and 2016 in the Netherlands. All estimates for transmission parameter $\beta$ were obtained using method A (see main text).

| **Farm identifier** | **Values for the latent period (days)** | $\boldsymbol{\beta}$ **MLE** | | | **Mean time of virus introduction (days)** | | |
| --- | --- | --- | --- | --- | --- | --- | --- |
|  |  | **Shorter latent period** | **Default latent period** | **Longer latent period** | **Shorter latent period** | **Default latent period** | **Longer latent period** |
| *Chickens 2014* |  |  |  |  |  |  |  |
| A | 1, 2, 3 | 2.3 | 5 | 11.1 | 13.6 | 14.8 | 16.1 |
| B | 1, 2, 3 | 8.2 | 34.4 | - | 8.9 | 9.8 | - |
| C | 1, 2, 3 | 2.2 | 4.4 | 15.1 | 10.6 | 11.8 | 12.3 |
| *Chickens 2016* |  |  |  |  |  |  |  |
| F | 0, 1, 2 | 2 | 8.5 | 28.1 | 5.3 | 7.4 | 9 |
| C | 0, 1, 2 | 2.2 | 10.9 | - | 3.9 | 5.9 | - |
| *Ducks 2016* |  |  |  |  |  |  |  |
| H | 0, 1, 2 | 0.8 | 1.6 | 3.3 | 12.3 | 14.5 | 15.9 |
| I | 0, 1, 2 | 2.1 | 11.8 | - | 7.7 | 9.5 | - |
| E | 0, 1, 2 | 0.6 | 0.95 | 1.5 | 16.1 | 18.8 | 20.9 |

**Table S13.** The maximum likelihood estimate for transmission parameter $\beta$ as function of the length of the infectious period of individuals dying from disease for outbreaks of highly pathogenic avian influenza of subtype H5N8 on Dutch poultry farms in 2014 and 2016 in the Netherlands. All estimates for transmission parameter $\beta$ were obtained using method A (see main text).

| **Farm identifier** | **Values for the infectious period (days)** | $\boldsymbol{\beta}$ **MLE** | | | **Time of virus introduction (days)** | | |
| --- | --- | --- | --- | --- | --- | --- | --- |
|  |  | **Shorter infectious period** | **Default infectious period** | **Longer infectious period** | **Shorter infectious period** | **Default infectious period** | **Longer infectious period** |
| *Chickens 2014* |  |  |  |  |  |  |  |
| A | 1, 2.5, 4 | 7.5 | 5 | 4.5 | 13.2 | 14.8 | 16.4 |
| B | 1, 2.5, 4 | - | 34.4 | 37 | - | 9.8 | 11.2 |
| C | 1, 2.5, 4 | 6.2 | 4.4 | 4.3 | 10.7 | 11.8 | 13.2 |
| *Chickens 2016* |  |  |  |  |  |  |  |
| F | 0.5, 1.1, 2 | 12.6 | 8.5 | 7.6 | 6.8 | 7.4 | 8.4 |
| C | 0.5, 1.1, 2 | 15.6 | 10.9 | 9.9 | 5.4 | 5.9 | 6.9 |
| *Ducks 2016* |  |  |  |  |  |  |  |
| H | 2, 3.5, 5 | 1.7 | 1.6 | 1.6 | 13.3 | 14.5 | 16 |
| I | 2, 3.5, 5 | 9.3 | 11.8 | - | 8.8 | 9.5 | - |
| E | 2, 3.5, 5 | 0.95 | 0.95 | 0.95 | 17.7 | 18.8 | 20.1 |

**Table S14.** The maximum likelihood estimate for transmission parameter $\beta$ as function of the length of the infectious period for recovering individuals for outbreaks of highly pathogenic avian influenza of subtype H5N8 on Dutch meat duck farms in 2016 in the Netherlands. All estimates for transmission parameter $\beta$ were obtained using method A (see main text).

| **Farm identifier** | **Values for the infectious period (days)** | $\boldsymbol{\beta}$ **MLE** | | | **Time of virus introduction (days)** | | |
| --- | --- | --- | --- | --- | --- | --- | --- |
|  |  | **Shorter infectious period** | **Default infectious period** | **Longer infectious period** | **Shorter infectious period** | **Default infectious period** | **Longer infectious period** |
| H | 4, 6, 8 | 1.7 | 1.6 | 1.6 | 14.4 | 14.6 | 14.5 |
| I | 4, 6, 8 | 11.8 | 11.8 | 11.8 | 9.5 | 9.5 | 9.5 |
| E | 4, 6, 8 | 1 | 0.95 | 0.95 | 19.2 | 18.8 | 18.8 |

**Table S15.** The maximum likelihood estimate for transmission parameter $\beta$ as function of the percentage of individuals that die for outbreaks of highly pathogenic avian influenza of subtype H5N8 on Dutch meat duck farms in 2016 in the Netherlands. All estimates for transmission parameter $\beta$ were obtained using method A (see main).

| **Farm identifier** | **Values for the percentage of animals dying from disease** | $\boldsymbol{\beta}$ **MLE** | | | **Time of virus introduction (days)** | | |
| --- | --- | --- | --- | --- | --- | --- | --- |
|  |  | **Lower mortality** | **Default mortality** | **Higher mortality** | **Lower mortality** | **Default mortality** | **Higher mortality** |
| H | 10, 20, 30 | 1.7 | 1.6 | 1.7 | 15.8 | 14.5 | 13.7 |
| I | 10, 20, 30 | - | 11.8 | 9.8 | - | 9.5 | 9.7 |
| E | 10, 20, 30 | 1 | 0.95 | 0.95 | 20.4 | 18.8 | 17.8 |

**Results S1: The percentage of simulated epidemics dying out in the initial stochastic phase**

**Table S16.** The percentage of simulated epidemics of highly pathogenic avian influenza of subtype H5N8 on Dutch poultry farms in 2014 and 2016, that was discarded, because the simulated epidemic died out before the observed disease-induced cumulative mortality was reached (Table 1 in the main text). Simulations were performed with transmission rate β set to the maximum likelihood estimate and to the boundaries of the 95% confidence interval for a given outbreak farm. The stochastic simulation model is described in Supplementary Methods S1. Additional details about the simulations and parameter settings of the model are given in the main text.

| **Farm identifier** | **Percentage of discarded simulations** | | |
| --- | --- | --- | --- |
|  | **β**^a^ **set to maximum likelihood estimate** | **β set to lower boundary of 95% CI** | **β set to upper boundary of the 95% CI** |
| *Chickens 2014^b^* |  |  |  |
| A | 0 | 0 | 0 |
| B | 0 | 0 | 0 |
| C | 0 | 0.8 | 0 |
| *Chickens 2016* |  |  |  |
| F | 0 | 0 | 0 |
| C | 0 | 0 | 0 |
| *Ducks 2016* |  |  |  |
| H | 0.1 | 0.8 | 0 |
| I | 0 | 0 | 0 |
| E | 0.4 | 28.4 | 0 |

^a^) The maximum likelihood estimate and 95% confidence bounds of parameter β are given in Table 4 of in the main text.

^b^) The settings of other epidemiological parameters are given in Table 3 of the main text.

**References**

1 Lloyd, A. L. Realistic Distributions of Infectious Periods in Epidemic Models: Changing Patterns of Persistence and Dynamics. *Theor. Popul. Biol.* **60**, 59-71 (2001).

2 Kang, H.-M. *et al.* Novel reassortant influenza A(H5N8) viruses among inoculated domestic and wild ducks, South Korea, 2014. *Emerging Infect. Dis.* **21**, 298-304 (2015).

3 Grund, C. *et al.* A novel European H5N8 influenza A virus has increased virulence in ducks but low zoonotic potential. *Emerg. Microbes Infect.* **7**, 132 (2018).

4 Bae, Y. *et al.* Pathological evaluation of natural cases of a highly pathogenic avian influenza virus, subtype H5N8, in broiler breeders and commercial layers in South Korea. *Avian Dis.* **59**, 175-182 (2015).

5 Bertran, K. *et al.* Lack of chicken adaptation of newly emergent Eurasian H5N8 and reassortant H5N2 high pathogenicity avian influenza viruses in the U.S. is consistent with restricted poultry outbreaks in the Pacific flyway during 2014-2015. *Virology* **494**, 190-197 (2016).

6 Gamoh, K. *et al.* Protective efficacy of stockpiled vaccine against H5N8 highly pathogenic avian influenza virus isolated from a chicken in Kumamoto prefecture, Japan, in 2014. *J. Vet. Med. Sci.* **78**, 139-142 (2016).

7 Kapczynski, D. R. *et al.* Vaccination with virus-like particles containing H5 antigens from three H5N1 clades protects chickens from H5N1 and H5N8 influenza viruses. *Vaccine* **34**, 1575-1581 (2016).

8 Kim, S. M. *et al.* Vaccine efficacy of inactivated, chimeric hemagglutinin H9/H5N2 avian influenza virus and its suitability for the marker vaccine strategy. *J. Virol.* **91** (2017).

9 Lee, D.-H. *et al.* Pathogenicity of the Korean H5N8 highly pathogenic avian influenza virus in commercial domestic poultry species. *Avian Pathol.* **45**, 208-211 (2016).

10 Song, B. M. *et al.* Pathogenicity of H5N8 virus in chickens from Korea in 2014. *J Vet Sci* **16**, 237-240 (2015).

11 Steensels, M. *et al.* Protection afforded by a recombinant Turkey herpesvirus-H5 vaccine against the 2014 European highly pathogenic H5N8 avian influenza strain. *Avian Dis.* **60**, 202-209 (2016).

12 Tanikawa, T. *et al.* Pathogenicity of H5N8 highly pathogenic avian influenza viruses isolated from a wild bird fecal specimen and a chicken in Japan in 2014. *Microbiol. Immunol.* **60**, 243-252 (2016).

13 Zeng, X. *et al.* Protective efficacy of an H5N1 inactivated vaccine against challenge with lethal H5N1, H5N2, H5N6, and H5N8 influenza viruses in chickens. *Avian Dis.* **60**, 253-255 (2016).

14 Tiensin, T. *et al.* Transmission of the highly pathogenic avian influenza virus H5N1 within flocks during the 2004 epidemic in Thailand. *J. Infect. Dis.* **196**, 1679-1684 (2007).

15 Bos, M. E. H. *et al.* Estimating the day of highly pathogenic avian influenza (H7N7) virus introduction into a poultry flock based on mortality data. *Vet. Res.* **38**, 493-504 (2007).

16 Diekmann, O. & Heesterbeek, J. A. P. *Mathematical epidemiology of infectious diseases*. (John Wiley & Sons, 2000).
